# Single-cell transcriptomics defines keratinocyte differentiation in avian scutate scales

**DOI:** 10.1038/s41598-021-04082-1

**Published:** 2022-01-07

**Authors:** Julia Lachner, Florian Ehrlich, Matthias Wielscher, Matthias Farlik, Marcela Hermann, Erwin Tschachler, Leopold Eckhart

**Affiliations:** 1 Department of Dermatology, Medical University of Vienna, Vienna, Austria; 2 Department of Medical Biochemistry, Medical University of Vienna, Vienna, Austria

**Keywords:** Evolutionary developmental biology, Differentiation, Intermediate filaments, Animal physiology

## Abstract

The growth of skin appendages, such as hair, feathers and scales, depends on terminal differentiation of epidermal keratinocytes. Here, we investigated keratinocyte differentiation in avian scutate scales. Cells were isolated from the skin on the legs of 1-day old chicks and subjected to single-cell transcriptomics. We identified two distinct populations of differentiated keratinocytes. The first population was characterized by mRNAs encoding cysteine-rich keratins and corneous beta-proteins (CBPs), also known as beta-keratins, of the scale type, indicating that these cells form hard scales. The second population of differentiated keratinocytes contained mRNAs encoding cysteine-poor keratins and keratinocyte-type CBPs, suggesting that these cells form the soft interscale epidermis. We raised an antibody against keratin 9-like cysteine-rich 2 (KRT9LC2), which is encoded by an mRNA enriched in the first keratinocyte population. Immunostaining confirmed expression of *KRT9LC2* in the suprabasal epidermal layers of scutate scales but not in interscale epidermis. Keratinocyte differentiation in chicken leg skin resembled that in human skin with regard to the transcriptional upregulation of epidermal differentiation complex genes and genes involved in lipid metabolism and transport. In conclusion, this study defines gene expression programs that build scutate scales and interscale epidermis of birds and reveals evolutionarily conserved keratinocyte differentiation genes.

## Introduction

Keratinocytes of the epidermis form a cornified cell layer at the body surface which protects against water loss and insults from the environments. In coordination with mesenchymal cells, epidermal keratinocytes also form skin appendages, such as claws, hair, feathers and scales, which have important functions in defense, capture of prey, thermoregulation and locomotion^1–4^. In birds most parts of the body surface are covered by a soft epidermis which suppresses water loss whereas hard skin appendages, such as the beak, feathers, and claws are used for interactions with the environment that require mechanical resilience^5^. The lower legs and the toes of birds are covered by scales which can be distinguished into scutate and reticulate scales^6,7^.

Scutate scales are located on the tarsometatarsus and on the dorsal side of the toes. They consist of overlapping hard scales that are separated by interscale or hinge regions. The structure of scutate scales resembles that of overlapping scales of reptiles. However, the hypothesis that avian scutate scales are homologous to reptilian scales, meaning that they have been inherited from a common ancestor of archosaurs (birds and crocodilians)^8–10^, has been challenged. The alternative hypothesis holds that avian scutate scales are secondarily derived from feathers^6,11,12^. Like feathers and scales of squamates, avian scutate scales develop from an anatomical placode^13–15^. The epidermal compartment of scutate scale placodes is characterized by the expression of beta-catenin (CTNNB1)^13^. A patterning mechanism distinct from that of avian scutate scales leads to the development of non-overlapping reticulate scales^7^.

The protective function of scutate scales depends on their structure and molecular composition. Corneous beta-proteins (CBPs), also known as beta-keratins^16^, keratins and other proteins were shown to be expressed in mature scutate scales^17–26^. A transcriptome analysis of embryonic scutate scales provided information on genome-wide gene expression, but only a subset of selected keratin intermediate filament and CBP genes were localized by mRNA in situ hybridization in hard scales and soft interscale regions^27^. To the best of our knowledge, a comprehensive gene expression catalog resolving the alternating pattern of soft and hard cornification has not been reported yet. In a recent study, we isolated keratinocytes from chicken leg skin, cultured them in an in vitro model of avian skin and determined their transcriptome^28^. Differentiation of keratinocytes in this culture system induced the expression of many genes, including members of the keratin family^29^, that were not expressed in monolayer cultures. However, it remained elusive which genes are expressed in the hard and soft segments of scutate scales in vivo.

Here, we report single-cell RNA-sequencing (scRNA-seq) of chicken leg skin and the characterization of two distinct types of differentiated keratinocytes of scutate scales.

## Results

### Keratin KRT9LC2 is a marker of differentiated keratinocytes in scutate scales of chickens

**Figure 1:**
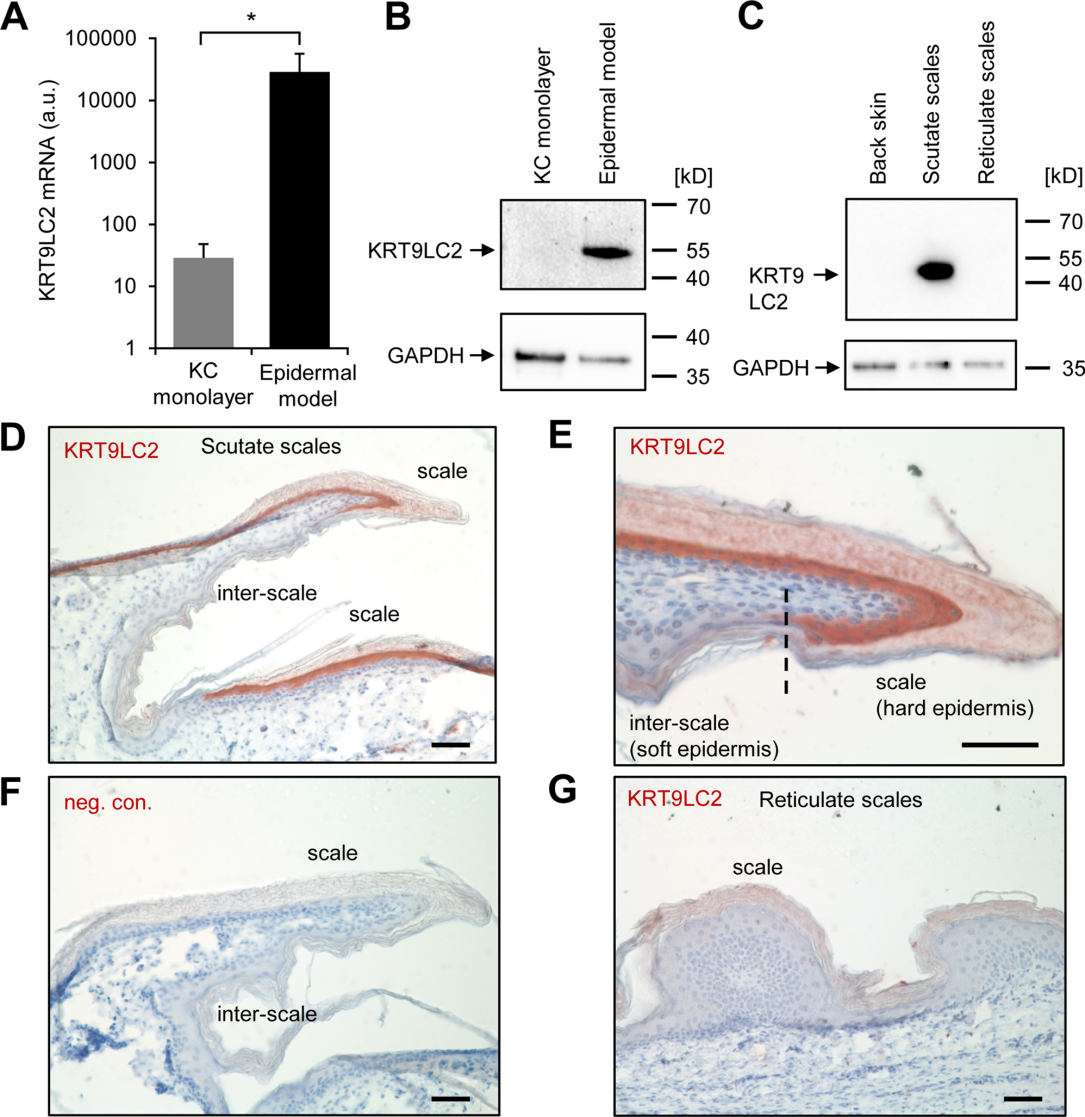
KRT9LC2 is a marker of hard scutate scales of chicken leg skin. (**A**) Quantitative RT-PCR analysis of *KRT9LC2* expression during differentiation of chicken leg keratinocytes (KC) in vitro. RNA was isolated from chicken KCs (n = 3, biological replicates) growing in monolayer culture and in an epidermal model. *KRT9LC2* mRNA was quantified by RT-PCR and normalized to the housekeeping gene *HMBS*. a.u., arbitrary units. (**B**,**C**) Western blot analysis of KRT9LC2 and GAPDH in cultured cells (**B**) and tissues of chicks (**C**). Bands at predicted sizes are indicated by arrows. The positions of molecular weight markers (kD, kilo-Dalton) are shown of the right. (**D**,**E**,**G**) Immunohistochemical detection of KRT9LC2 (red) in scutate scales (**D**,**E**) and reticulate scales (**G**). A negative control (neg. con.) staining in which the anti-KRT9LC2 antiserum was replaced by non-immune serum is shown in panel (**F**). Scale bars, 50 µm.

The chicken has a diversified set of keratins^29^ which are hypothesized to mark specific states of epithelial cell differentiation. Keratin 9-like cysteine-rich 2 (KRT9LC2), also referred to as Hard Acid Sauropsid-specific 2 (HAS2)^29^, was detected by RT-PCR in scutate scales and analysis of transcriptome data^28^ suggested that it is transcriptionally upregulated during in vitro differentiation of keratinocytes isolated from chicken leg skin (Fig. 1A). An antibody raised against a carboxy-terminal peptide of the KRT9LC2 protein (Supplementary Fig. S1) detected a prominent band at the predicted size of 51 kD in protein extracts from the stratified epidermis of an in vitro skin model but not in extracts from monolayer cultures of undifferentiated chicken keratinocytes (Fig. 1B). The KRT9LC2 protein was also detected in extracts from scutate scales but not in back skin or reticulate scales of chickens (Fig. 1C).

Immunohistochemical staining yielded a strong KRT9LC2 signal in the suprabasal keratinocytes of scutate scales whereas interscale epidermis was not immunostained (Fig. 1D,E). When the primary antibody was replaced by preimmune serum, there was no immunostaining, confirming the absence of unspecific staining (Fig. 1F). The reticulate scales were immunonegative (Fig. 1G). These results demonstrated that KRT9LC2 is a marker of differentiated keratinocytes that form the hard outer surface of scutate scales.

### scRNA-seq analysis reveals two distinct populations of differentiated keratinocytes in chicken scutate scales

**Figure 2:**
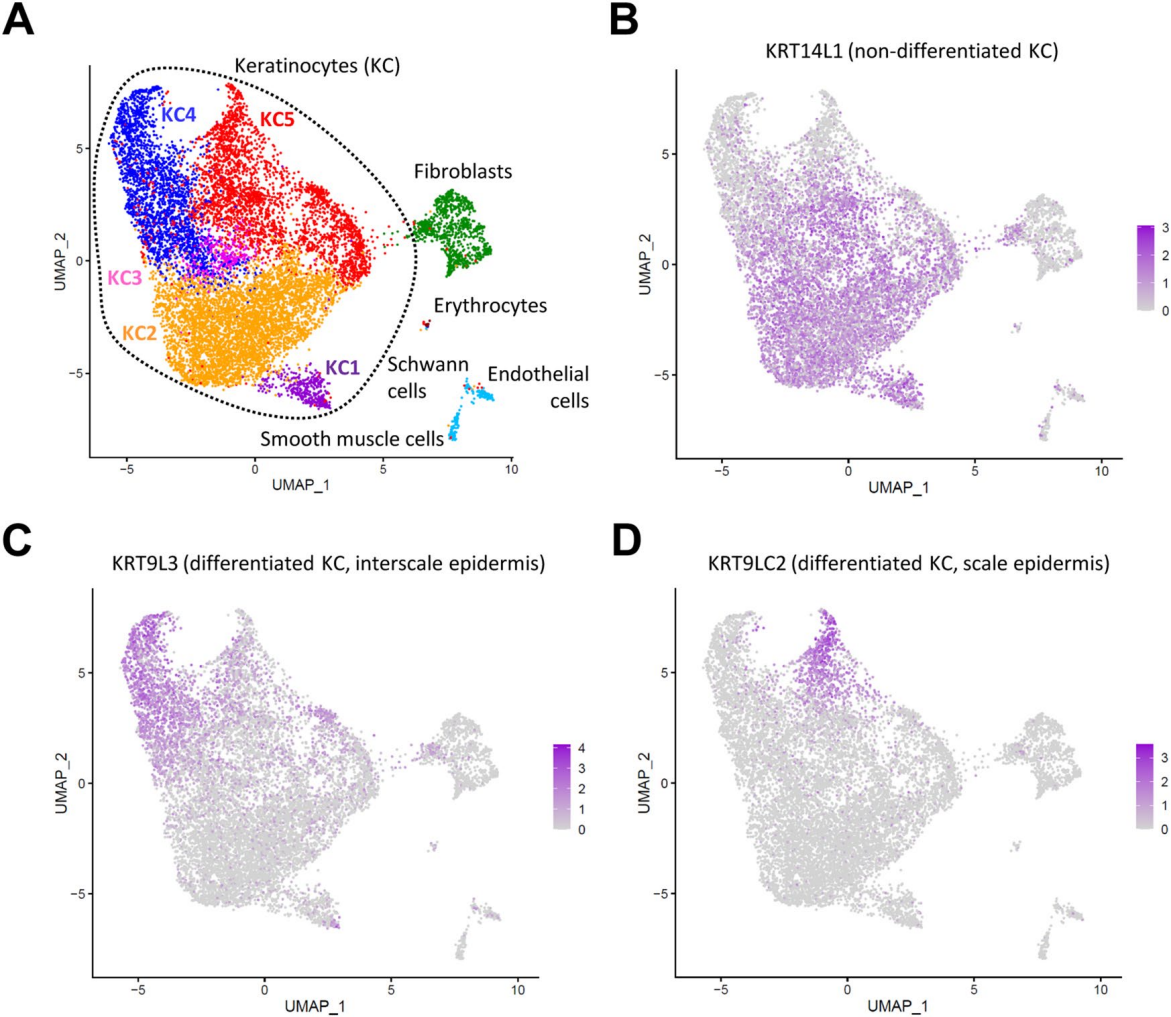
Single-cell RNA-sequencing (scRNA-seq) analysis of scutate scales of chicks. (**A**) Uniform manifold approximation and projection (UMAP) of cells from chicken scutate scales according to similarity of their transcriptome. Unsupervised clustering resulted in 8 clusters that are indicated by different colors. Cell types were identified by the expression of specific markers, so that 5 unsupervised clusters (KC1-KC5) could be defined as keratinocytes. (**B–D**) Feature plots showing the expression level of *KRT14L1* (**B**), *KRT9L3* (**C**) and *KRT9LC2* (**D**) in each cell depicted in UMAP plots. The expression levels are color-coded from grey (no expression) to purple (high expression level).

To characterize gene expression during keratinocyte differentiation in chicken scutate scales, we isolated cells from the legs of 1-day old chicks (n = 3) and subjected them to single-cell RNA-sequencing (scRNA-seq). The protocol was designed to enrich for epidermal keratinocytes but smaller populations of fibroblasts, smooth muscle cells, endothelial cells, Schwann cells and erythrocytes were also detected (Fig. 2A, Supplementary Fig. S2).

According to nearest neighbor clustering as implemented in Seurat^30^, keratinocytes were divided into 5 clusters (Supplementary Table S1). Clusters KC1, KC2 and KC3 (Fig. 2A) represented non-differentiated cells, characterized by high expression levels of *KRT14L1* (Fig. 2B) ^29^, which is the avian homolog of *KRT14*, a marker of the basal epidermal layer in mammals. *KRT9L3* (Fig. 2C), a cysteine-poor keratin upregulated during differentiation of chicken keratinocytes in in vitro skin models^28^, was expressed at high levels in cluster KC4 (Fig. 2A), for which CBP63 was defined as another marker gene (Supplementary Table S1). Expression of *CBP63* (GenBank Gene ID: 101751614), previously referred to as “β-keratin, Chr25, Ktn13” ^27^, was demonstrated by mRNA in situ hybridization in the interscale epidermis of scutate scales ^27^. Therefore, cluster KC4 contained keratinocytes of the soft interscale epidermis. Cluster KC5 (Fig. 2A) was defined by marker genes such as *KRT9LC2* (Supplementary Table S1; Fig. 2D). From the immunolocalization of *KRT9LC2* (Fig. 1D) we inferred that *KRT9LC2*-positive cells represented the hard scales. The clustering of cells was reproduced in the 3 biological replicates investigated in this study (Supplementary Fig. S3).

### Keratinocyte differentiation is associated with the expression of distinct genes in scale and interscale segments of chicken scutate scales

**Figure 3:**
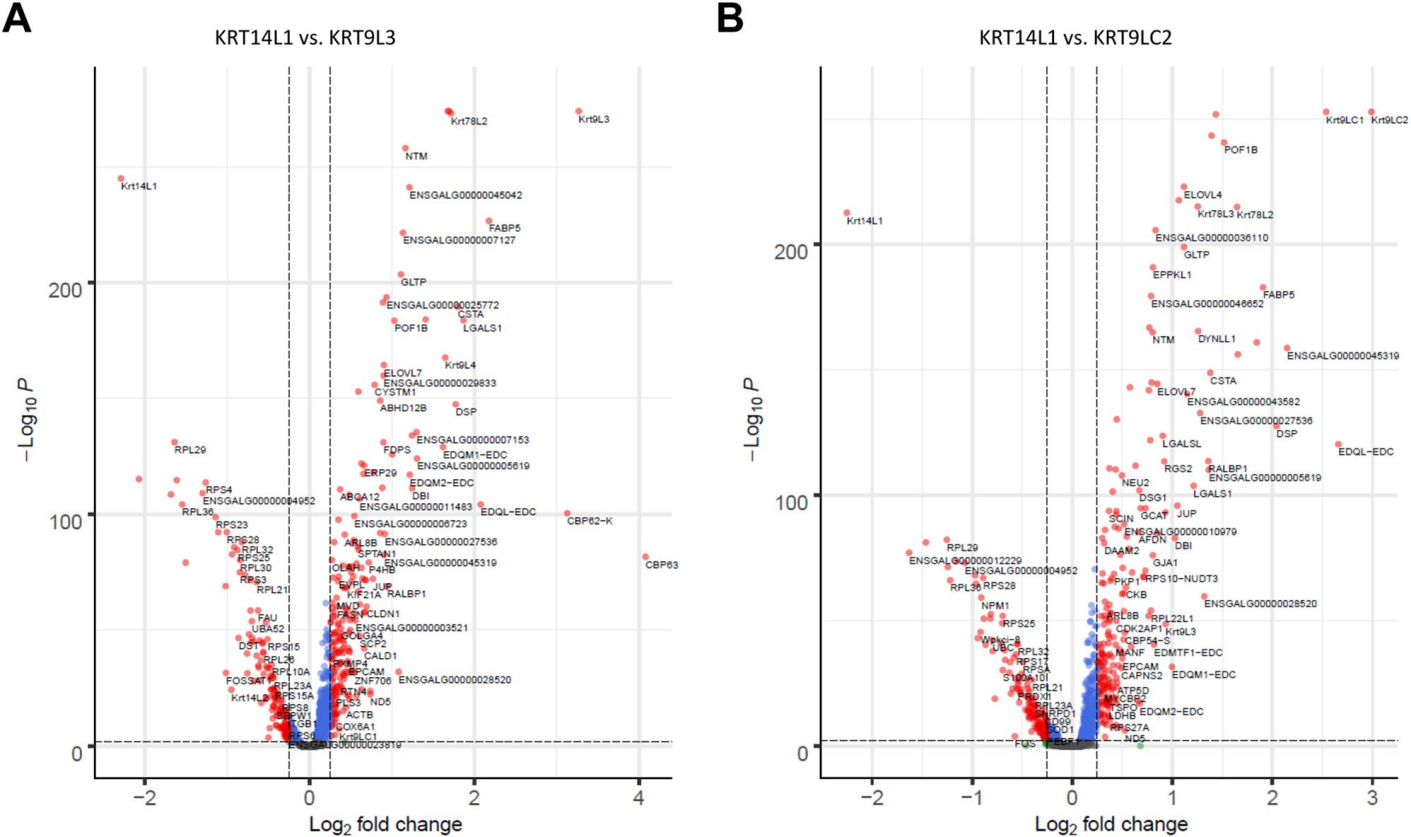
Volcano plots of genes differentially expressed in *KRT9L3*-high versus *KRT14L1*-high and *KRT9LC2*-high versus *KRT14L1*-high cells. Significance (-log10 of adjusted *P* value) was plotted against Log2 of Fold-change of gene expression levels in *KRT9L3*-positive versus *KRT14L1*-positive keratinocytes (**A**) and *KRT9LC2*-positive versus *KRT14L1*-positive keratinocytes (**B**).

To determine genes that are upregulated during keratinocyte differentiation in scutate scales, we compared gene expression in cells containing *KRT14L1* transcripts, marking the non-differentiated state of keratinocytes, versus gene expression in cells positive for one or both of the two differentiation markers defined above, i.e. *KRT9L3* and *KRT9LC2*. In *KRT9L3*-positive cells 219 genes were expressed at higher levels than in *KRT14L1*-positive cells (Fig. 3A, Supplementary Table S2), and in *KRT9LC2*-positive cells 213 genes were upregulated (> 0.25 Log2-fold average upregulation, *P* < 0.001) (Fig. 3B, Supplementary Table S3). The majority of these genes (n = 133), including the type II keratin, *KRT78L2*, the epidermal differentiation complex gene *EDQL* (Supplementary Fig. S4), homologs of mammalian keratinocyte differentiation-associated genes, such as *DSP*, *FABP5*, *POF1B*, and others (Supplementary Tables S2 and S3) were upregulated both in *KRT9L3*-positive and in *KRT9LC2*-positive cells relative to *KRT14L1*-positive cells.

To identify specific markers of hard and soft epidermal differentiation in scutate scales, we compared gene expression levels in *KRT9LC2*-positive versus *KRT9L3*-positive cells and determined the genes that differed most strongly with regard to their expression in these cells (Tables 1 and 2, Supplementary Fig. S4). *KRT9LC2*-positive cells accumulated, amongst others, the cysteine-rich keratin *KRT9LC1* (Supplementary Fig. S4B), scale-associated CBPs such as *CBP53* (Supplementary Fig. S4F), the lectin *LGASL1*, and *HOPX*, whose mammalian ortholog regulates keratinocyte differentiation^31^ (Table 1). Likewise, *CTNNB1*, previously reported as a regulator of scutate scale development, was found enriched in differentiated keratinocytes of hard scales (Table 1). *KRT9L3*-positive cells accumulated cysteine-poor keratin *KRT9L4* (Supplementary Fig. S4A), keratinocyte-associated CBPs such as *CBP62* and *CBP63* (Supplementary Fig. S4E), the lectin *LGAS1*, and *PRDX1*, an antioxidant enzyme^32^ (Table 2). Thus, the results of this study suggest catalogues of genes associated with keratinocyte differentiation in hard epidermal segments (Table 1) and genes associated with keratinocyte differentiation in soft interscale segments (Table 2) of chicken scutate scales.

**Table 1:** Gene expression levels in KRT9L3 + versus KRT9LC2 + cells: Genes upregulated in KRT9LC2 + keratinocytes (hard scales).

Rank	Gene symbol	Gene name	*P* value (adjusted)	Average Log(e) FC	Percentage (KRT9L3 + cells)	Percentage (KRT9LC2 + cells)
1	KRT9LC2	Keratin 9-like cysteine-rich 2	1.50E−130	− 1.76	14	100
2	KRT9LC1	Keratin 9-like cysteine-rich 1	1.76E−102	− 1.50	11	90
3	ENSGALG00000036110	Serine/threonine-protein kinase ULK4	4.12E−46	− 0.57	4	49
4	ENSGALG00000046632	Ly6/PLAUR domain-containing protein 2-like	2.17E−42	− 0.74	16	65
5	CBP53-S	Corneous beta-protein 53 scale type	9.59E−32	− 0.52	3	37
6	MT4	Metallothionein 4	1.81E−26	− 0.82	21	57
7	LGALSL	Galectin like	1.04E−21	− 0.45	33	65
8	LMO7	LIM domain 7	6.79E−21	− 0.37	22	56
9	ENSGALG00000010979	Hydroxysteroid 17-beta dehydrogenase 11	2.24E−20	− 0.31	14	46
10	ENSGALG00000027083	Pancreatic lipase-related protein 2	4.60E−19	− 0.28	4	28
11	ENSGALG00000027207	PERP2, TP53 apoptosis effector	1.72E−18	− 0.41	98	99
12	ENSGALG00000039470	60S ribosomal protein L10-like 1	1.47E−17	− 0.23	100	100
13	CDK2AP1	Cyclin dependent kinase 2 associated protein 1	4.98E−17	− 0.28	14	44
14	RPS12	Ribosomal protein S12	4.77E−16	− 0.38	96	99
15	PSMD10	Proteasome 26S subunit, non-ATPase 10	1.03E−15	− 0.24	7	31
16	CTNNB1	Catenin beta 1	6.79E−15	− 0.34	43	72
17	RORA	RAR related orphan receptor A	1.22E−14	− 0.31	22	52
18	ENSGALG00000028451	Metallothionein 4-like	2.83E−14	− 0.56	72	84
19	KRT14L2	Keratin 14-like 2	5.95E−13	− 0.35	7	29
20	ENSGALG00000029833	Digestive cysteine proteinase 2-like	1.16E−12	− 0.37	62	80
21	EXOC6	Exocyst complex component 6	4.07E−12	− 0.16	2	19
22	CBP55-S	Corneous beta-protein 55 scale type	8.40E−12	− 0.21	1	15
23	TFPI2	Tissue factor pathway inhibitor 2	3.27E−11	− 0.22	7	27
24	HOPX	HOP homeobox	6.03E−11	− 0.29	35	60
25	ENSGALG00000023818	Heat shock protein family B (small) member 9	1.70E−10	− 0.51	48	72
26	ENSGALG00000020078	H3 histone, family 3C	3.34E−10	− 0.26	83	91
27	CBP52L-S	Corneous beta-protein 52-like scale type	4.33E−09	− 0.18	0	12
28	ENSGALG00000040260	Tubulin alpha 1c	1.02E−08	− 0.18	8	27
29	BOK	BCL2 family apoptosis regulator BOK	1.07E−08	− 0.20	10	30
30	RPS15	Ribosomal protein S15	1.66E−08	− 0.19	100	100
31	METAP2	Methionyl aminopeptidase 2	4.60E−08	− 0.24	33	55
32	TPT1	Tumor protein, translationally-controlled 1	4.84E−08	− 0.21	99	99
33	ENSGALG00000027536	PERP1, TP53 apoptosis effector	6.27E−08	− 0.25	84	90
34	SEPP1	Selenoprotein P	6.59E−08	− 0.20	9	27
35	TUBB3	Tubulin beta 3 class III	6.60E−08	− 0.16	5	22
36	RPL4	Ribosomal protein L4	9.82E−08	− 0.24	94	99
37	RPL15	Ribosomal protein L15	1.04E−07	− 0.21	97	99
38	CRIP2	Cysteine rich protein 2	1.18E−07	− 0.17	10	28
39	CBP54-S	Corneous beta-protein 54 scale type	1.49E−07	− 0.38	1	11
40	PAK1	p21 (RAC1) activated kinase 1	2.46E−07	− 0.16	7	24
41	SERPINB2	Serpin family B member 2	2.57E−07	− 0.22	10	29
42	PDE6D	Phosphodiesterase 6D	2.82E−07	− 0.13	3	16
43	EDMTF1-EDC	Epidermal differentiation protein MTF1, EDC	3.00E−07	− 0.58	1	12
44	ENSGALG00000036099	Eukaryotic translation elongation factor 1 delta	3.62E−07	− 0.21	93	95
45	GCAT	Glycine C-acetyltransferase [Homo sapiens	3.71E−07	− 0.23	43	63
46	PTTG1IP	PTTG1 interacting protein	4.62E−07	− 0.25	21	40
47	SPINK6	Serine peptidase inhibitor Kazal type 6	5.15E−07	− 0.53	3	16
48	MSX2	Msh homeobox 2	5.63E−07	− 0.12	2	13
51	LGR4	Leucine-rich repeat G protein-coupled receptor 4	1.52E−06	− 0.16	6	22
57	ENSGALG00000045796	Cytosolic phospholipase A2 epsilon-like	2.61E−06	− 0.12	1	12

**Table 2:** Gene expression in KRT9L3 + versus KRT9LC2 + cells: Genes upregulated in KRT9L3 + keratinocytes (interscale epidermis).

Rank	Gene symbol	Gene name	P-value (adjusted)	Average Log(e) FC	Percentage (KRT9L3 + cells)	Percentage (KRT9LC2 + cells)
1	KRT9L3	Keratin 9-like 3	1.50E−129	1.76	100	46
2	ENSGALG00000007127	Fatty acid desaturase 1 (FADS1)	5.13E−38	0.69	57	12
3	CBP63-K	Corneous beta-protein 63 keratinocyte type	1.89E−34	2.47	63	26
4	ENSGALG00000045042	D-beta-hydroxybutyrate dehydrogenase, mito.-like	4.14E−33	0.64	63	22
5	CBP62-K	Corneous beta-protein 62 keratinocyte type	8.51E−29	2.02	52	16
6	GAPDH	Glyceraldehyde-3-phosphate dehydrogenase	4.79E−27	0.48	95	82
7	KRT9L4	Keratin 9-like 4	9.55E−27	1.08	40	6
8	LGALS1	Galectin 1	2.21E−26	0.46	100	99
9	PRDX1	Peroxiredoxin 1	4.20E−26	0.54	58	21
10	S100A6	S100 calcium binding protein A6	5.43E−24	0.60	97	88
11	GPX1	Glutathione peroxidase 1	5.47E−24	0.38	100	94
12	IL13RA2	Interleukin 13 receptor subunit alpha 2	1.01E−20	0.28	28	1
13	SCCPDH	Saccharopine dehydrogenase (putative)	2.76E−20	0.43	53	21
14	ENSGALG00000021451	Uncharacterized oxidoreductase-like	4.21E−20	0.39	58	25
15	ENSGALG00000045989	Trypsin II-P29-like, lincRNA	5.95E−19	0.41	49	18
16	S100A11	S100 calcium binding protein A11	2.50E−18	0.40	91	81
17	ENSGALG00000007220	Ferritin heavy chain 1	1.84E−17	0.41	56	23
18	ST13	ST13 Hsp70 interacting protein	8.37E−16	0.29	45	15
19	ACAT2	Acetyl-CoA acetyltransferase 2	9.12E−14	0.29	35	10
20	BARX2B	BARX homeobox 2B	1.42E−13	0.25	20	1
21	YBX1	Y-box binding protein 1	1.84E−13	0.35	86	62
22	PPA1	Inorganic pyrophosphatase 1	6.03E−12	0.22	30	7
23	MOGAT1	Monoacylglycerol O-acyltransferase 1	5.43E−11	0.19	19	2
24	OLAH	Oleoyl-ACP hydrolase	7.13E−11	0.18	19	1
25	ATP5G3	ATP synthase, mitochondrial F0 complex, subunit C3	3.76E−10	0.37	60	38
26	ANXA1	Annexin A1	4.20E−10	0.34	46	20
27	NAP1L1	Nucleosome assembly protein 1 like 1	4.79E−10	0.26	52	26
28	TKT	Transketolase	9.13E−10	0.21	31	10
29	DUSP14	Dual specificity phosphatase 14	1.55E−09	0.27	41	18
30	EDQM2-EDC	Epidermal differentiation protein Q motif 2, EDC	1.61E−09	0.39	35	12
31	ENSGALG00000045170	Lymphocyte antigen 6E-like	2.82E−09	0.31	20	3
32	CHCHD2	Coiled-coil-helix-coiled-coil-helix domain containing 2	3.27E−09	0.31	76	62
33	FDPS	Farnesyl diphosphate synthase	5.30E−09	0.45	40	18
34	ENSGALG00000006723	Isopentenyl-diphosphate delta isomerase 1	5.48E−09	0.28	34	13
35	IL20RA	Interleukin 20 receptor subunit alpha	6.03E−09	0.16	19	3
36	PPDPF	Pancreatic progenitor cell diff. proliferation factor	6.45E−09	0.29	65	46
37	ENSGALG00000008439	CD36	9.64E−09	0.20	23	5
38	HOMER2	Homer scaffold protein 2	2.80E−08	0.19	28	8
39	ACLY	ATP citrate lyase	3.09E−08	0.26	38	17
40	ATP5G1	ATP synthase, H + transporting, mito. F0 compl. sub. C1	4.27E−08	0.29	57	35
41	HACD3	3-hydroxyacyl-CoA dehydratase 3	5.08E−08	0.20	32	12
43	EDQM1-EDC	Epidermal differentiation protein Q motif 1, EDC	8.39E−08	0.47	39	18
45	KRT9L1	Keratin 9-like 1	1.14E−07	0.17	15	1
52	HSD17B12	Hydroxysteroid 17-beta dehydrogenase 12	4.27E−07	0.21	35	15
54	ELOVL4	ELOVL fatty acid elongase 4	6.89E−07	0.41	76	62
74	FASN	Fatty acid synthase	8.33E−05	0.17	26	10
75	ENSGALG00000027494	Serine palmitoyltransferase small subunit B (SPTSSB)	1.05E−04	0.19	40	21
79	LOR1-EDC	Loricrin 1, EDC	2.53 E−04	0.17	10	1
80	ENSGALG00000045194	Lipase member M-like 2	2.53 E−04	0.10	10	1
92	ENSGALG00000044962	Arylacetamide deacetylase-like 4-like (AADAC4L)	2.55E−03	0.21	21	8

## Discussion

Differentiation of keratinocytes underlies the growth of epithelial skin structures, such as claws, hair, feathers and scales of mammals, reptiles and birds. The molecular control of keratinocyte differentiation is well characterized for mammalian interfollicular epidermis and skin appendages^33–35^, whereas little is known about the genetic regulation of keratinocyte differentiation in sauropsids. The results of the present study shed light into keratinocyte differentiation in scutate scales and provide a basis for the comparative analysis of further epithelial cell differentiation processes in avian claws, beak and feathers.

We have used scRNA-seq to characterize two types of keratinocyte differentiation, leading to the hard outer surface and the soft interscale epidermis of scutate scales. The methodology was adapted from successful scRNA-seq analyses of human and mouse skin^36–39^. scRNA-seq of mouse tail skin revealed two paths of keratinocyte differentiation into scale and interscale epidermis^40^. Of note, hard scales of the mouse tail were found to contain transcripts of cysteine-rich keratins such as *KRT31* whereas the soft interscale regions contained epidermal keratins such as *KRT10* and epidermal differentiation complex (EDC) genes such as involucrin^40,41^. In contrast to the availability of many antibodies against mouse keratinocyte proteins, we had only one antibody, anti-KRT9LC2, specific for a keratin expressed in chicken scutate scales. This was a significant limitation of the present study. We were able to ascertain the expression of KRT9LC2 in differentiated keratinocytes of hard scales, and mRNA in situ hybridization data published by Wu et al. 2015 supported the expression of CBP63 in interscale epidermis^27^. However, other putative differentiation markers, that are suggested by our results, remain to be localized in situ in future studies.

Gene expression in interscale epidermis of chicken leg skin showed several similarities to gene expression in two models of chicken epidermis with a soft cornified layer^28^. scRNA-seq analysis of chicken back skin and bulk RNA-seq analysis of an organotypic model of chicken skin revealed expression of EDC genes and cysteine-poor but not cysteine-rich keratins^28^. Many of the genes upregulated during differentiation of back skin keratinocytes^28^, such as *KRT9L4*, *LOR1*, *KRT9L3*, *BDH1L*, *EDQM2*, *SPTSSB*, *EDQM1*, *AADACL4B*, *LIPML2*, and *ELOVL4* (Table 2) were enriched in interscale versus scale epidermis. Conversely, genes enriched in hard scale epidermis, such as *KRT9LC2*, *KRT9LC1*, *CBP53-S*, *CBP54-S*, *CBP55-S*, *EDMTF1*, and *MT4* (Table 1) were not upregulated during differentiation of back skin keratinocytes^28^. Therefore, we conclude that the genetic program of keratinocyte differentiation in the soft interscale epidermis of scutate scales is similar to the keratinocyte differentiation program in the soft back epidermis.

Keratinocyte differentiation in the hard outer surface of scutate scales differs substantially from that in the interscale regions. The results of the present study establish *KRT9LC2*, also referred to as HAS2 keratin^29^, as a protein marker of the hard scutate scales and identify other genes that are co-regulated with *KRT9LC2*. Among these scale-associated genes was *KRT9LC1* (GenBank Gene ID:772,080) (Supplementary Fig. S3B), also referred to as Hard Acidic Sauropsid-specific 1 (HAS1) keratin^29^. In situ hybridization of transcripts corresponding to this gene (then named *KRT13A*) demonstrated predominant expression in the outer surface epithelium of scutate scales^27^, thus validating our scRNA-seq data. Another gene co-expressed with *KRT9LC2* was *CBP55-S*, a scale CBP (beta-keratin). In the aforementioned study of Wu and colleagues^27^, expression of this gene (then named β-keratin, Chr25, Scale18) was detected by in situ hybridization specifically in the outer surface epithelium of scutate scales, further validating our scRNA-seq data.

An important result of this study is the genome-wide gene expression catalog of scutate scale epidermis resolved at the single-cell level. In addition to the genes discussed above, many more genes with differentiation-dependent expression were identified both in soft interscale epidermis (Supplementary Table S2) and hard scales (Supplementary Table S3). With regard to ongoing efforts to characterize evolutionarily ancient and derived patterns of gene expression during epidermal keratinocyte differentiation^28,29,42–48^, the results of the present study support the hypothesis that EDC genes, anti-inflammatory interleukin 1 family cytokines (IL-36RN and IL-1RN) (Supplementary Tables S2 and S3; Supplementary Fig. S5) and lipid metabolism and lipid transport-related genes, such as *FABP5* and *GLTP*^28^ belong to the common keratinocyte differentiation program of amniotes. The transcriptome data generated in this study will be particularly useful for characterizing the process of hard cornification in a non-mammalian model species. The single-cell transcriptomes of chicken leg skin are now accessible for data searches according to criteria not limited to keratinocyte differentiation, so that new research questions pertaining to avian skin biology can be addressed in future studies.

## Methods

### Tissue preparation and scRNA-seq analysis

One day old chicks (strain Lohmann) were obtained from Schropper GmbH, Gloggnitz, Austria. Skin was excised from the leg of sacrificed animals and incubated in thermolysin (0.5 mg/ml) (Sigma-Aldrich) at 37 °C for 45 min. The lower dermis was removed using forceps and the remaining tissue, including the epidermis and parts of the upper dermis, was processed further according to protocols established for human skin^39,49^. For the isolation of cells, the tissue was split into two fractions that were incubated either in buffer-enzyme mix of the Whole Skin Dissociation Kit human (MACS Milteny Biotech) for 2.5 h at 37 °C or with 0.05% trypsin/EDTA (Thermo Fisher Scientific) and DNase 1 (10 µg/ml) (Roche Diagnostics) at 37 °C for 15 min. Afterwards the samples were combined and processed according to the manufacturer’s protocol (Whole Skin Dissociation Kit human, MACS Milteny Biotech). In brief, the epidermis-enzyme mix was diluted in 0.5 ml medium and dissociated with the gentleMACS Dissociator. The ground tissue was filtered through 100-micron (Falcon) and 40-micron (Falcon) meshes. Subsequently, cells were stained with DAPI dye for 10s and viable cells were sorted via an AriaFusion high-speed cell sorting device (BD Biosciences, San Jose, CA, USA). Single cell RNA sequencing was performed according to a published protocol^49^. In brief, a 10× Genomics Chromium instrument (10× Genomics, Pleasanton, CA) was used for single cell partitioning and barcoding and Illumina HiSeq 3000/4000 (Illumina, San Diego, CA) was used for sequencing (Center for Molecular Medicine, Vienna, Austria). Using the CellRanger Fastq pipeline (10X Genomics) the demultiplexed raw sequencing data were aligned to the chicken reference genome Gallus_gallus-5.0.

### Analysis of scRNA-sequencing data

We distinguished between background noise and droplets containing cells using emptyDrops^50^. Briefly, this method models ambient RNA background in the data set and tests for deviations from the background RNA. We used a false discovery rate of 0.05 to call cells to be included into further analysis. On the other end of the spectrum we used scran package to remove droplets containing more than one cell^51^. The applied approach simulates thousands of doublets by adding together two randomly chosen single cell profiles. For the doublet score calculation, cell clustering including the set randomly generated doublets is performed. Then for each cell of the original dataset, the number of simulated doublets in their neighbourhood is recoded and used as input for score calculation. We used 200 nearest neighbours for each cell and applied a threshold of doublet score > 4 to identify doublets in each dataset separately. Doublet score was log10 of the ratio between simulated doublet cells and total number of neighbours taken into consideration for each cell. The data obtained from 3 biological replicates were submitted to the NCBI Gene Expression Omnibus (GEO) database under accession numbers GSE179690 (BioProject PRJNA744554). The individual samples were referred to as “leg skin 1” (BioSample: SAMN20109848, SRA: SRX11375855), “leg skin 2” (BioSample: SAMN20109847, SRA: SRX11375856) and “leg skin 3” (BioSample: SAMN20109846, SRA: SRX11375857).

Subsequent to individual quality control of samples, raw read counts of across datasets were combined to one count matrix. Reads from 11,779 cells were included in the final analysis. We used Pearson residuals derived from a generalized negative binomial model of UMI counts as input for principal component analysis and differential gene expression analysis. This approach is implemented in the R package Seurat as sctransform^52^. Apart from cellular sequencing depth, which is added by default to the regression model, we also adjusted for mitochondrial RNA content, batch, and cell cycle score. Calculation of cell cycle scores was performed as implemented in Seurat package where gene expression of cell cycle marker genes are combined to score. The score consisted of 43 genes primarily expressed in G1/S and 55 primarily expressed in G2/M^53^. We removed cells with a mitochondrial RNA content above 15%. Batch correction between individual datasets was done as part of the principal component analysis using the Harmony algorithm^54^. Harmony starts off with user supplied information about batch, then uses fuzzy clustering to assign each cell to multiple clusters in a way that batch diversity in each cluster is maximized. To get a correction factor for each cell, global and batch-specific centroids are calculated for each cluster. Data are corrected for batch and the procedure is repeated until convergence of global and batch-specific centroids^54^. We used PC 1–15 for subsequent UMAP analysis and nearest neighbor based clustering at a resolution of 0.2.

For further analysis of differentially expressed genes (DEGs), three different clusters, characterized by a certain expression threshold of marker genes, e.g. KRT9L3 > 2, KRT9LC2 > 1.5 and KRT14L1 > 1.5, were created. Gene expression levels in different clusters were compared and adjusted *P* values were calculated according to the standard algorithm implemented in Seurat^30^.

### Quantitative reverse-transcription polymerase chain reaction

RNA was isolated from chicken tissues and skin models^28^ and purified with TriFast according to a published protocol^46^ and reverse-transcribed with the iScriptTM cDNA synthesis kit (Biorad, Hercules, CA). Polymerase chain reactions (PCRs) were performed with primer pairs specific for *KRT9LC2* (KRT9LC2-s, 5′-GAATGCCGCTACAACAACCAC-3′ and KRT9LC2-a, 5′-TGCTTCAGGGATCTCTCATTG-3′), *IL1RN* (IL1RN-s, 5′-GAGAAGGTGTTTTGGGTGCC-3′ and IL1RN-a, 5′-TAGGTGCGGAAGAAGGTGAA-3′), *IL36RN* (IL36RN-s, 5′-GAGCTCAGCCGTACCACTAC-3′ and IL36RN-a, 5′-AACAGCTTCACCTCCTCCAG-3′), and the housekeeping gene *Hydroxymethylbilane synthase* (*HMBS*) (HMBS-s, 5′-AACTGTGGGAAAACGCTCAG-3′ and HMBS-a, 5′-TTCTCTTCAGTCCAGCAGCA-3′) on a Roche LightCycler with LC480 SYBR Green I Master Kit according to the manufacturer’s protocol. Quantitative analysis of *IL1RN* and *IL36RN* expression in chicken tissues was performed according to a published method^46^. The expression levels of these genes were compared between scutate scales and other tissues, considering differences with a *P* value of < 0.05 significant (two-sided t-test).

### Western blot analysis

Proteins were prepared from chicken skin and scales by treatment with the Precellys system (VWR, International, Radnor, PA) and from chicken keratinocytes cultured in vitro^28^ by sonication in Laemmli buffer containing 2% SDS. Thirty µg of protein per lane were electrophoresed through an ExcelGel SDS 8–18% polyacrylamide gel (GE Healthcare Life Sciences) and afterwards blotted onto a nitrocellulose membrane (GVS Life Sciences). Subsequently, the membrane was blocked with phosphate-buffered saline containing 5% milk powder (Sigma-Aldrich), 2% bovine serum albumin (Sigma-Aldrich) and 0.1% Tween (Sigma-Aldrich) at room temperature for one hour, and incubated with mouse anti-KRT9LC2 antibody (1:500) that was raised in mice by immunization with a synthetic peptide CAAAEIQVPCRRICD, corresponding to the carboxy-terminus of the protein (GenBank accession number XP_418162.6, GenBank definition: keratin, type I cytoskeletal 19) (Supplementary Fig. S1) conjugated to keyhole limpet hemocyanin, according to a published protocol ^55^. After overnight incubation at 4 °C, the membrane was washed and sheep anti-mouse immunoglobulin G (1:10,000, GE Healthcare UK Limited) coupled with horseradish peroxidase used as secondary antibody at room temperature for one hour. The chemiluminescence system (Clarity Western ECL Substrate, BioRad) served for the protein detection. For loading control, the membrane was reincubated with anti-mouse GAPDH (1:5000, HyTest) and sheep anti-mouse immunoglobulin G (1:10,000, GE Healthcare UK Limited), coupled with horseradish peroxidase. The recordings of the chemiluminescence signal over the entire blots are shown in Supplementary Fig. S6 and the relevant portions thereof are depicted in Fig. 1C,D.

### Immunohistochemistry

Immunohistochemistry was performed according to published protocols with modifications^46^. In brief, chicken tissue samples were fixed in 7.5% formaldehyde and embedded in paraffin. Citrate buffer pH6 (DAKO) was used to retrieve the antigens and mouse anti-KRT9LC2 antibody (1:500) as primary antibody. To block unspecific binding, 10% sheep serum was added to secondary sheep anti-mouse immunoglobulin G (GE Healthcare), and further the nuclei were counterstained with haematoxylin. For control experiments, the primary antibody was replaced by the pre-immune serum.

### Ethics statement

All animal procedures were approved by the Animal Care and Use Committee of the Medical University of Vienna. All procedures were performed in accordance with the guidelines established by this committee and in adherence to the ARRIVE guidelines ^56^.

## Supplementary Information

Supplementary Information.

## Data Availability

Single-cell transcriptomes generated in this study are available at GEO under accession number GSE179690. All other data generated or analysed during this study are included in this published article and its Supplementary Information files.
