# Supplementary material for: Single-cell transcriptomics defines keratinocyte differentiation in avian scutate scales
Source: Sci Rep. 2022 Jan 7;12:126. doi: 10.1038/s41598-021-04082-1 (PMC8742010; doi:10.1038/s41598-021-04082-1)
Supplement: Supplementary file 1 — Supplementary Information. [file 41598_2021_4082_MOESM1_ESM.pdf]

Supplementary Data: Supplementary Figures and Tables

Single-cell transcriptomics defines keratinocyte differentiation in avian scutate scales

Julia Lachner, Florian Ehrlich, Matthias Wielscher, Matthias Farlik, Marcela Hermann, Erwin Tschachler, Leopold Eckhart

Content

Supplementary Figures S1-S6

Supplementary Tables S1-S3

>KRT9LC2
MSSVSSGRYTTCGIGGSRGFSGRSYCGGVNYGGGLSSGSLVGGSYGGGLGAAVLGGCSGIGFSGGSARFG
GGIGGGLGISLGGGVVGGGFSGDGILLSGDEKVTMQNLNDRLASYLDKVRCLEQENADLECRIREWYAKQ
GPFCEPRDYSCYYKEIEDLQNQIVCATIDNNKIILNIDNSRMTADDFRVKYETELALRQSVEADINGLRQ
VLDQLTLCRSDLEAQLESLREELCCLKKNHEEEMNCLRKQSTGDVSVEVNACPGPDLRKILEEMRCQYET
LIERNRKEVEDWYECKIEEVNREVITSGQEVETCNNQVTELRRQLQALEIDLQAQLSQRDNLESSLAETE
CRYNNHLGELQTQITCVEQQLADLRAEMECQNQEYKILLDVKCRLEQEIHTYRCLLEGGQQDLIQQGGIG
QLSGLGGGVARTGGIGGGGIIRTSHTYTSSAQMPSCAAAEIQVPCRRICD

Supplementary Figure S1. Amino acid sequence of chicken KRT9LC2 and epitope of anti-KRT9LC2. The amino acid sequence of chicken KRT9LC2 (GenBank accession number XP_418162.6) is shown in FASTA format. Cysteine residues are highlighted by yellow shading. The intermediate filament domain is underlined. The epitope of the antiserum against KRT9LC2 is indicated by a red box.

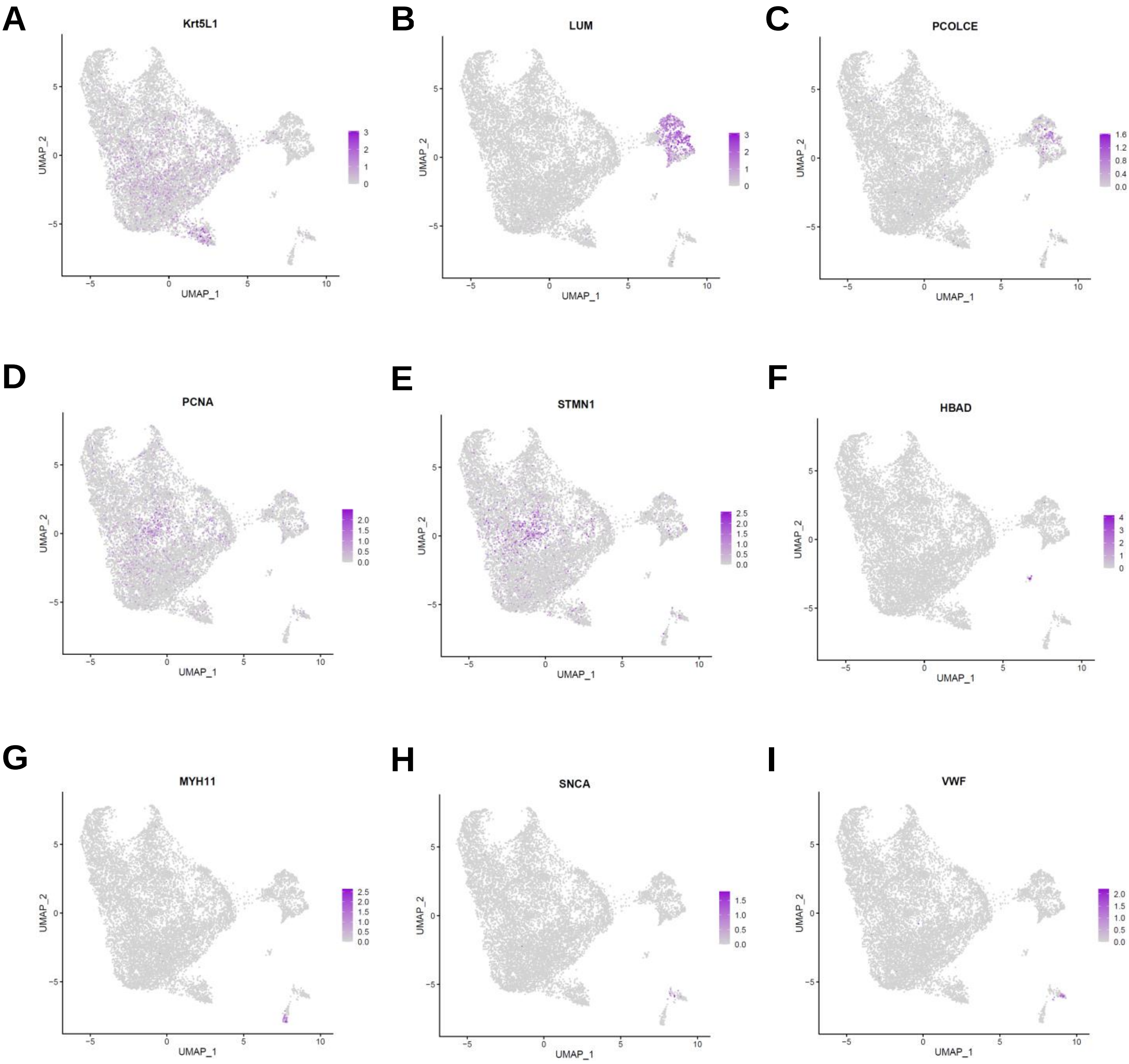

Supplementary Figure S2. Feature plots of markers. The expression levels of KRT5L1 (A), LUM (B), PCOLCE (C), PCNA (D), STMN1 (E), HBAD (F), MYH1 (G), SNCA (H) and VWF (I) are color-coded from grey (no expression) to purple (high expression level) in each cell depicted in UMAP plots.

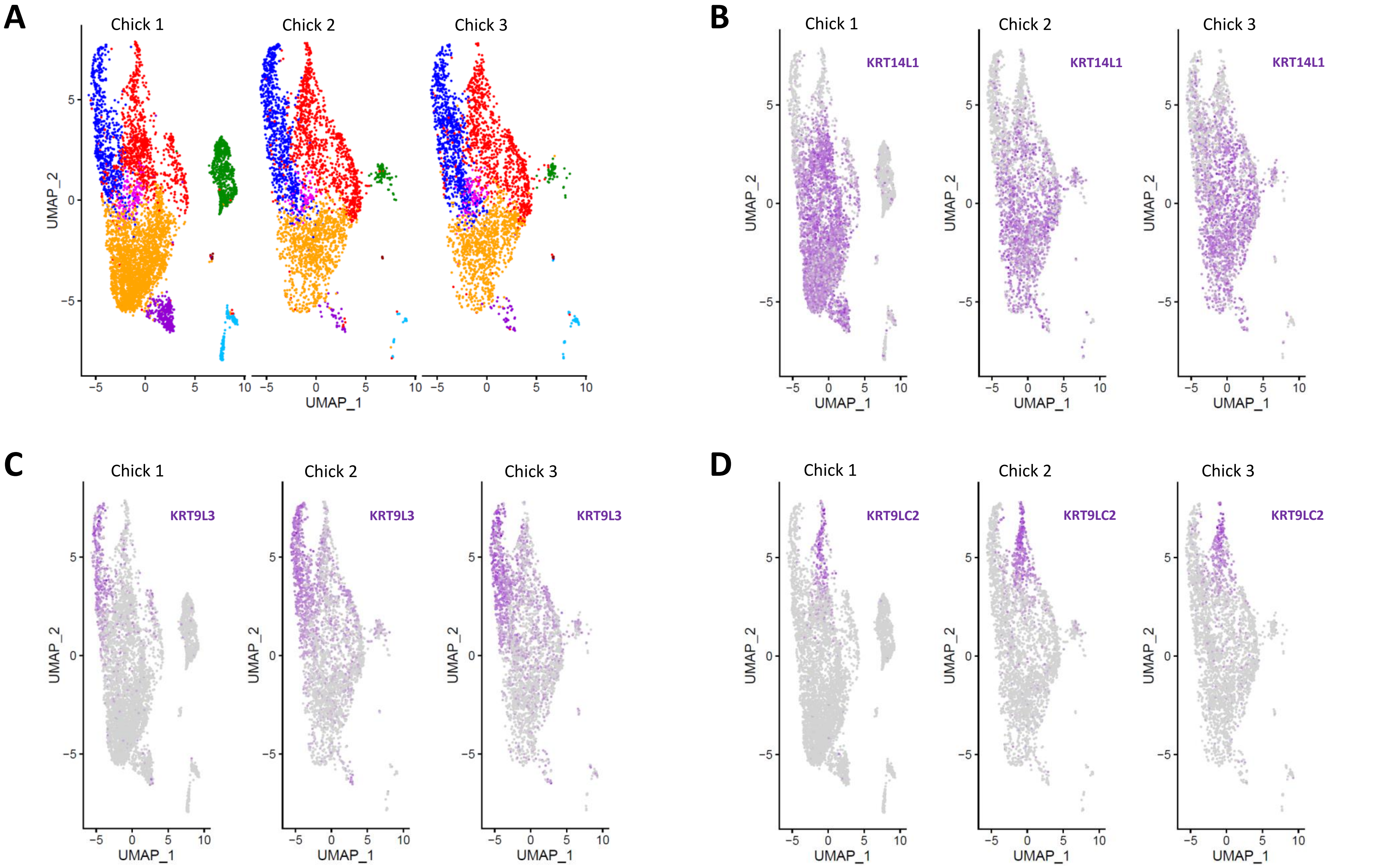

Supplementary Figure S3. scRNA-seq analysis of biological replicates. scRNA-seq was performed on 3 independent biological samples (chick 1, 2 and 3). UMAP plots of unbiased cell clustering (A) and expression levels of KRT14L1 (B), KRT9L3 (C) and KRT9LC2 (D) confirmed a high degree of reproducibility. The color codes are the same as in Figure 2.

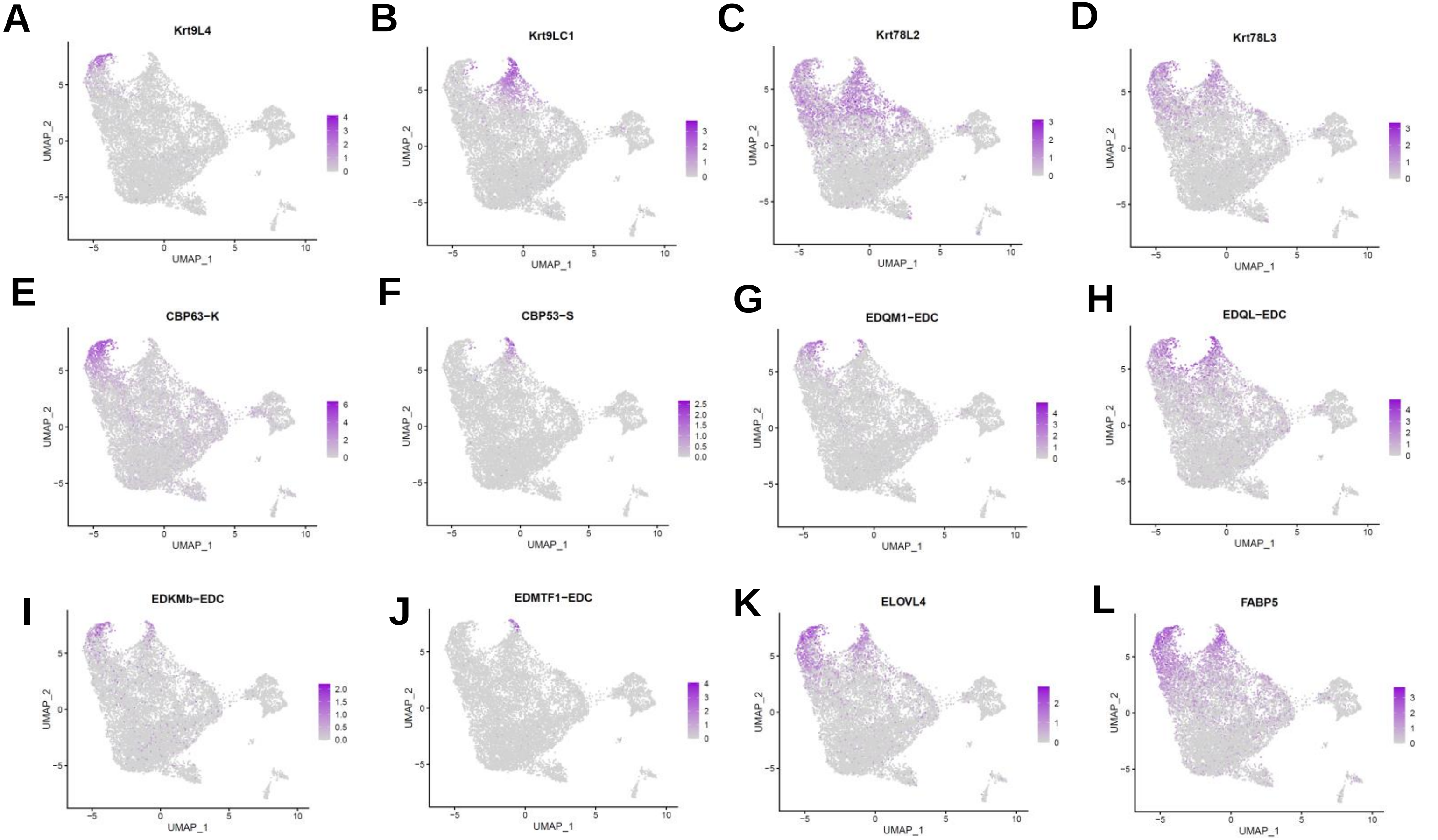

Supplementary Figure S4. Feature plots of keratinocyte differentiation-associated genes in chicken scutate scales. The expression levels of KRT9L4 (**A**), KRT9LC1 (**B**), KRT78L2 (**C**), KRT78L3 (**D**), CBP63-K (**E**), CBP53-S (**F**), EDQM1 (**G**), EDQL (**H**), EDKM (**I**), EDMTF1 (**J**), ELOVL4 (**K**), and FABP5 (**L**) are color-coded from grey (no expression) to purple (high expression level) in each cell depicted in UMAP plots.

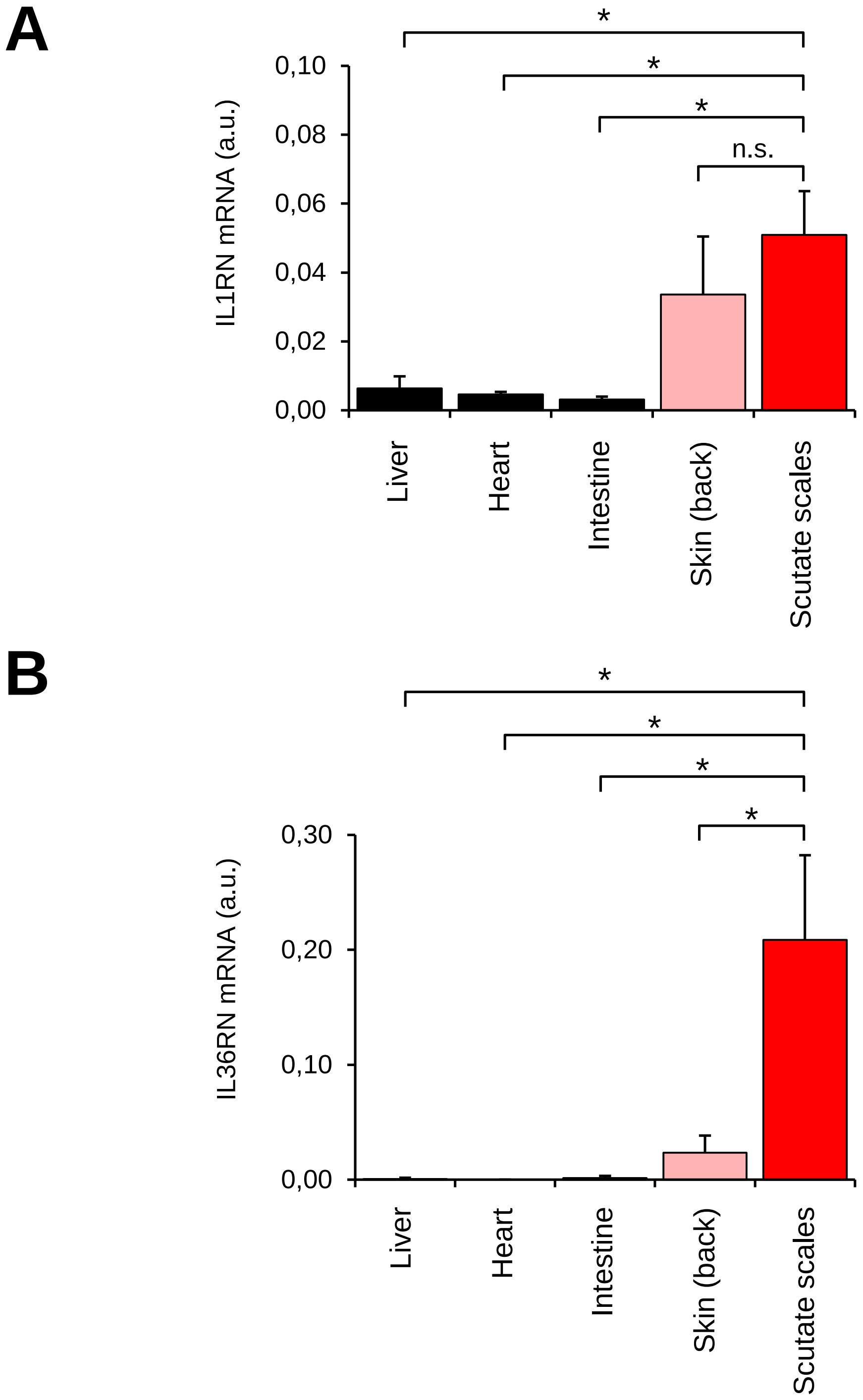

Supplementary Figure S5. RT-PCR analysis of *IL1RN* and *IL36RN* expression in scutate scales in comparison to other tissues of chickens. mRNAs of *IL1RN* (A) and *IL36RN* (B) were quantified by RT-PCR analysis of RNA preparations from tissues of n=3 chickens. The amounts of the target mRNAs are shown relative to mRNA of the house-keeping gene *HMBS*. The expression levels in scutate scales were compared to those in other tissues. Significant differences (P<0.05, 2-sided t-test) are indicated by asterisks. a.u., arbitrary units; n.s., not significant.

Western blots shown in figure 1B

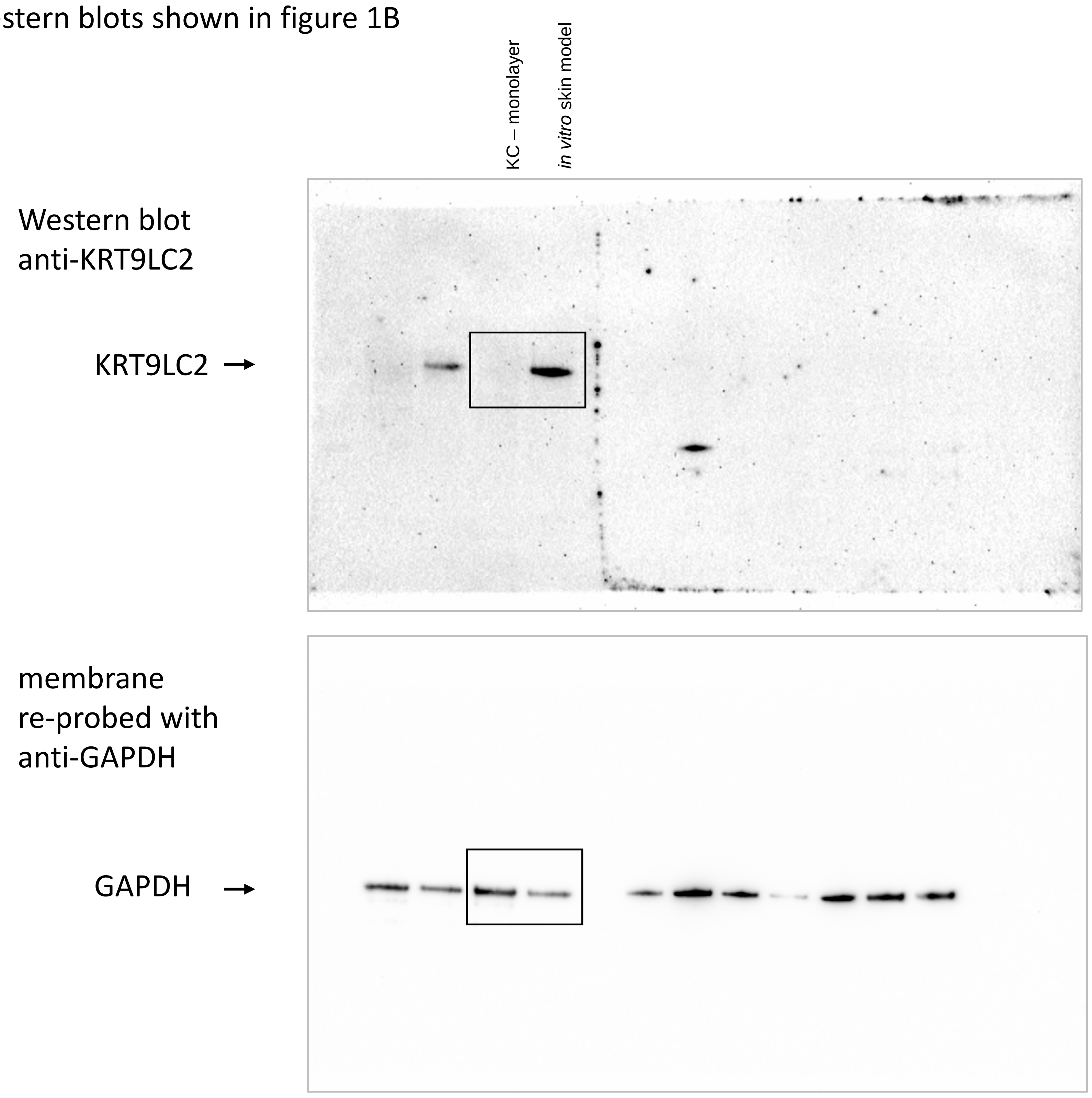

Western blots shown in figure 1C

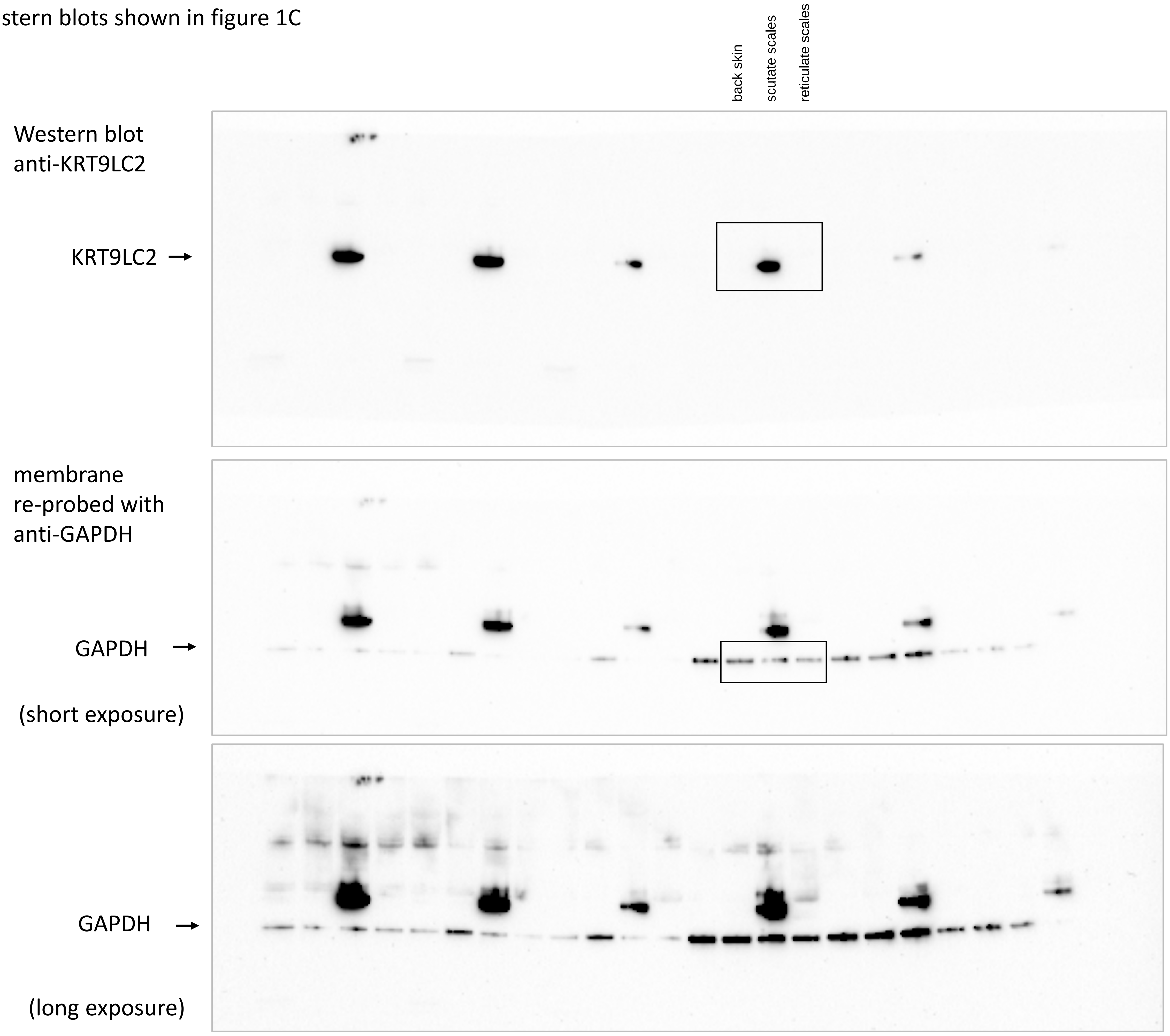

Supplementary Figure S6. Full-size Western blots which are displayed in cropped form in figure 1. Expression of KRT9LC2 was analyzed by Western blot. Subsequently, the membranes were re-probed with an antibody against GAPDH. The recordings of the chemiluminescence signal over the entire blots are shown. The relevant portions of the blots (marked by black rectangles) are displayed in Figure 1C, D.

Supplementary Table S1. Cluster markers

Cell cluster	Gene	P-value (adjusted)	Average Log(e) FC	pct,1	pct,2
KC5	MT4	0,0E+00	0,79	0,519	0,177
KC5	ENSGALG00000028451	0,0E+00	0,78	0,924	0,701
KC5	KRT9LC2	0,0E+00	0,77	0,338	0,049
KC5	DSP	3,8E-253	0,73	0,845	0,623
KC5	KRT14L2	2,5E-247	0,72	0,396	0,132
KC5	ATP6	8,3E-187	0,69	0,954	0,958
KC5	COX3	8,4E-216	0,69	0,993	0,987
KC5	KRT9LC1	0,0E+00	0,66	0,273	0,031
KC5	ENSGALG00000005619	2,4E-296	0,63	0,754	0,413
KC5	ND5	3,1E-176	0,61	0,868	0,829
KC5	EDQL-EDC	6,7E-106	0,60	0,362	0,173
KC5	ENSGALG00000027207	7,5E-250	0,56	0,842	0,581
KC5	ND6	2,2E-104	0,51	0,888	0,878
KC5	KRT78L2	4,8E-238	0,48	0,447	0,158
KC5	RALBP1	1,3E-160	0,47	0,560	0,322
KC5	ENSGALG00000045319	3,0E-55	0,47	0,220	0,106
KC5	JUP	1,4E-197	0,43	0,636	0,368
KC5	ENSGALG00000007153	1,8E-139	0,41	0,636	0,412
KC5	ND1	1,8E-78	0,40	0,868	0,870
KC5	LGALS1	1,7E-293	0,40	0,997	0,914
KC5	ENSGALG00000029833	4,5E-164	0,34	0,337	0,121
KC5	ENSGALG00000046632	1,4E-212	0,33	0,227	0,041
KC5	NFKBIA	2,1E-62	0,31	0,741	0,608
KC5	GJA1	6,4E-133	0,31	0,431	0,212
KC5	ANXA2	2,2E-128	0,30	0,897	0,753
KC5	DBI	4,1E-110	0,30	0,694	0,494
KC5	ENSGALG00000015140	1,2E-109	0,30	0,445	0,248
KC5	ENSGALG00000027536	2,9E-92	0,29	0,555	0,368
KC5	POF1B	2,0E-114	0,28	0,266	0,100
KC5	KLF6	1,3E-52	0,27	0,573	0,436
KC5	CTNNB1	1,6E-105	0,26	0,444	0,245
KC5	CLDN1	1,6E-97	0,26	0,470	0,274
KC5	MAFB	6,3E-86	0,26	0,528	0,343
KC5	DYNLL1	2,4E-111	0,26	0,366	0,170
KC5	FABP5	3,3E-103	0,26	0,486	0,262
KC4	CBP63-K	8,0E-184	1,91	0,511	0,268
KC4	CBP62-K	1,4E-186	1,36	0,373	0,134
KC4	KRT9L3	0,0E+00	1,14	0,719	0,208
KC4	LGALS1	0,0E+00	0,94	0,999	0,920
KC4	FABP5	0,0E+00	0,82	0,647	0,239
KC4	CSTA	0,0E+00	0,77	0,568	0,184
KC4	EDQM1-EDC	1,4E-117	0,58	0,188	0,048
KC4	ENSGALG00000005619	4,4E-234	0,58	0,765	0,435
KC4	DBI	7,6E-228	0,56	0,762	0,493
KC4	KRT9L4	1,1E-191	0,55	0,178	0,024
KC4	S100A6	7,5E-248	0,54	0,990	0,941
KC4	EDQL-EDC	1,5E-110	0,53	0,385	0,181
KC4	ELOVL4	3,3E-264	0,52	0,368	0,091
KC4	EDQM2-EDC	9,9E-115	0,49	0,167	0,038
KC4	ENSGALG00000027207	2,1E-235	0,47	0,867	0,594
KC4	ENSGALG00000045042	0,0E+00	0,46	0,363	0,055
KC4	ENSGALG00000043582	2,1E-174	0,42	0,272	0,069
KC4	ENSGALG00000007153	7,9E-166	0,42	0,684	0,417
KC4	KRT78L3	6,8E-229	0,42	0,356	0,093
KC4	NTM	1,3E-262	0,39	0,386	0,099
KC4	KRT78L2	2,6E-164	0,38	0,450	0,179

Supplementary Table S1 (continued). Cluster markers

Cell cluster	Gene	P-value (adjusted)	Average Log(e) FC	pct,1	pct,2
KC4	CALML3	3,0E-170	0,36	0,897	0,711
KC4	GPX1	1,2E-171	0,36	0,984	0,906
KC4	ENSGALG00000027536	2,3E-147	0,36	0,626	0,365
KC4	ANXA2	3,0E-153	0,34	0,932	0,755
KC4	GAPDH	4,7E-148	0,33	0,966	0,876
KC4	ENSGALG00000007127	1,8E-201	0,33	0,254	0,053
KC4	LDHB	2,4E-115	0,33	0,536	0,320
KC4	RPL22L1	4,0E-94	0,32	0,736	0,580
KC4	GLTP	1,3E-157	0,31	0,373	0,136
KC4	S100A11	9,1E-117	0,31	0,960	0,885
KC4	CD9	9,3E-213	0,31	0,325	0,083
KC4	ENSGALG00000021451	3,2E-166	0,30	0,404	0,154
KC4	TMEM254	2,4E-121	0,30	0,200	0,052
KC4	DYNLL1	3,6E-122	0,29	0,400	0,177
KC4	SCCPDH	1,0E-155	0,28	0,312	0,100
KC4	PRDX1	3,8E-68	0,28	0,556	0,396
KC4	P4HB	1,2E-84	0,27	0,441	0,254
KC4	ENSGALG00000045989	2,1E-119	0,26	0,325	0,128
KC4	KCTD1	2,4E-91	0,25	0,477	0,273
KC4	FDPS	1,1E-135	0,25	0,190	0,042
KC3	TUBA1A	7,2E-96	1,06	0,873	0,526
KC3	ENSGALG00000001475	5,8E-258	1,05	0,683	0,101
KC3	HMGB1	1,3E-111	0,84	0,817	0,346
KC3	H2AFV	1,8E-86	0,77	0,870	0,483
KC3	SMC2	0,0E+00	0,67	0,473	0,018
KC3	HMGN2	4,0E-117	0,62	0,701	0,207
KC3	TOP2A	0,0E+00	0,60	0,456	0,022
KC3	PCNA	1,4E-148	0,57	0,544	0,101
KC3	TUBB	3,7E-58	0,55	0,515	0,185
KC3	UBE2C	2,7E-299	0,52	0,361	0,019
KC3	C11orf31	1,3E-70	0,51	0,678	0,273
KC3	MT4	2,4E-30	0,51	0,538	0,256
KC3	ENSGALG00000006326	6,5E-55	0,50	0,825	0,489
KC3	HMGB2	3,3E-92	0,48	0,417	0,088
KC3	ENSGALG00000040586	4,4E-134	0,46	0,349	0,044
KC3	YBX1	8,1E-45	0,43	0,941	0,806
KC3	CHCHD2	1,8E-38	0,43	0,873	0,637
KC3	CDK1	0,0E+00	0,43	0,361	0,018
KC3	ATP5G3	2,6E-38	0,42	0,802	0,505
KC3	ENSGALG00000037167	1,4E-74	0,41	0,257	0,041
KC3	BIRC5	2,9E-232	0,41	0,408	0,034
KC3	NUCKS1	1,1E-34	0,41	0,568	0,271
KC3	SOD1	4,7E-66	0,40	0,556	0,188
KC3	ENSGALG00000014023	8,6E-52	0,38	0,506	0,181
KC3	ENSGALG00000028600	5,5E-48	0,38	1,000	0,980
KC3	ENSGALG00000000137	5,5E-45	0,37	0,660	0,306
KC3	MRPL51	2,0E-55	0,36	0,509	0,175
KC3	PTMA	2,0E-36	0,36	0,607	0,296
KC3	ENSGALG00000016442	0,0E+00	0,35	0,287	0,010
KC2	DST	0,0E+00	0,53	0,594	0,204
KC2	COL14A1	0,0E+00	0,47	0,572	0,185
KC2	ATIC	0,0E+00	0,44	0,608	0,218
KC2	ENSGALG00000004952	8,0E-178	0,37	0,982	0,949
KC2	ENSGALG00000012229	2,6E-65	0,37	0,835	0,832
KC2	KRT14L1	1,0E-243	0,35	0,838	0,487
KC2	TPT1	2,1E-233	0,32	0,998	0,994

Supplementary Table S1 (continued). Cluster markers

Cell cluster	Gene	P-value (adjusted)	Average Log(e) FC	pct,1	pct,2
KC2	ENSGALG00000023818	1,1E-67	0,32	0,865	0,811
KC2	ENSGALG00000016691	2,3E-176	0,32	0,793	0,572
KC2	BSG	3,2E-223	0,31	0,531	0,236
KC2	ZNF593	1,5E-166	0,29	0,753	0,527
KC2	UBC	6,6E-163	0,29	0,871	0,733
KC2	ENSGALG00000001634	7,4E-283	0,28	0,999	0,998
KC2	FAU	1,4E-249	0,28	0,999	0,991
KC2	NPM1	9,3E-134	0,27	0,776	0,563
KC1	ENSGALG00000036119	0,0E+00	1,00	0,700	0,036
KC1	ENSGALG00000033586	0,0E+00	0,92	0,673	0,031
KC1	ENSGALG00000034870	0,0E+00	0,85	0,617	0,028
KC1	ENSGALG00000037942	0,0E+00	0,80	0,571	0,033
KC1	ENSGALG00000043546	0,0E+00	0,65	0,469	0,022
KC1	KRT5L1	1,2E-49	0,62	0,397	0,134
KC1	ENSGALG00000038514	0,0E+00	0,59	0,442	0,030
KC1	FOS	7,3E-33	0,48	0,938	0,737
KC1	KRT14L1	8,0E-28	0,42	0,839	0,617
KC1	ND1	7,4E-39	0,41	0,973	0,866
KC1	UBC	1,3E-29	0,40	0,909	0,783
KC1	ENSGALG00000041527	0,0E+00	0,35	0,298	0,010
KC1	ENSGALG00000034566	6,8E-248	0,34	0,279	0,014
KC1	ATP1B3	8,2E-18	0,32	0,584	0,397
KC1	ND6	2,6E-17	0,32	0,973	0,878
KC1	BSG	1,2E-09	0,26	0,507	0,347
KC1	DUSP1	7,8E-03	0,26	0,614	0,537
KC1	ENSGALG00000041175	5,2E-171	0,26	0,239	0,016
FB	ENSGALG00000036073	0,0E+00	3,54	0,831	0,077
FB	COL1A2	0,0E+00	3,50	0,841	0,123
FB	COL3A1	0,0E+00	2,82	0,827	0,101
FB	APOA1	2,2E-208	2,12	0,379	0,075
FB	MGP	4,0E-76	2,04	0,426	0,206
FB	GSN	0,0E+00	1,75	0,640	0,112
FB	DCN	0,0E+00	1,70	0,840	0,308
FB	ENSGALG00000026613	0,0E+00	1,57	0,631	0,064
FB	ENSGALG00000032220	0,0E+00	1,54	0,601	0,019
FB	COL6A1	0,0E+00	1,50	0,749	0,024
FB	SPARC	0,0E+00	1,48	0,861	0,410
FB	GPX3	0,0E+00	1,37	0,652	0,041
FB	LUM	0,0E+00	1,33	0,657	0,016
FB	S100A10	4,1E-285	1,29	0,740	0,275
FB	COL5A1	0,0E+00	1,23	0,741	0,113
FB	VIM	0,0E+00	1,20	0,702	0,051
FB	COL6A2	0,0E+00	1,18	0,673	0,016
FB	COL6A3	0,0E+00	1,12	0,669	0,014
FB	WIF1	0,0E+00	1,11	0,383	0,013
FB	C2orf40	0,0E+00	1,10	0,603	0,020
FB	ENSGALG00000045199	0,0E+00	1,02	0,646	0,060
FB	SERPINF1	0,0E+00	1,01	0,534	0,016
FB	COL5A2	0,0E+00	0,85	0,599	0,038
FB	TGFBI	0,0E+00	0,84	0,453	0,010
FB	FN1	0,0E+00	0,76	0,468	0,025
FB	CTGF	0,0E+00	0,72	0,308	0,014
FB	ENSGALG00000036847	0,0E+00	0,72	0,429	0,009
FB	ENSGALG00000030886	0,0E+00	0,72	0,272	0,011
FB	SDC2	0,0E+00	0,68	0,487	0,029
FB	PMP22	0,0E+00	0,66	0,516	0,081

Supplementary Table S1 (continued). Cluster markers

Cell cluster	Gene	P-value (adjusted)	Average Log(e) FC	pct,1	pct,2
FB	FSTL1	0,0E+00	0,65	0,460	0,020
FB	COL8A1	0,0E+00	0,64	0,358	0,006
FB	ENSGALG00000041031	0,0E+00	0,58	0,313	0,005
FB	OLFML3	0,0E+00	0,57	0,411	0,007
FB	CCDC80	0,0E+00	0,57	0,397	0,007
ERY	HBAA	0,0E+00	4,11	1,000	0,003
ERY	HBAD	0,0E+00	3,23	1,000	0,002
ERY	H1F0	0,0E+00	2,13	0,964	0,002
ERY	CA2	0,0E+00	1,88	0,857	0,008
ERY	B2M	1,7E-39	1,19	0,786	0,088
ERY	BCL2L1	6,0E-12	0,56	0,464	0,078
ERY	NFE2L2	4,8E-24	0,54	0,429	0,038
ERY	RFESD	0,0E+00	0,50	0,321	0,000
ERY	NCOA4	7,2E-03	0,44	0,321	0,078
ERY	BPGM	7,3E-04	0,43	0,286	0,056
ERY	LAMP1	4,1E-02	0,41	0,393	0,123
ERY	BACH1	4,1E-02	0,36	0,214	0,042
ERY	HBE	0,0E+00	0,25	0,179	0,000
SMC/SC/EC	C11orf96	1,8E-144	1,45	0,570	0,094
SMC/SC/EC	COL4A1	0,0E+00	1,44	0,744	0,027
SMC/SC/EC	TAGLN	3,8E-292	1,21	0,260	0,006
SMC/SC/EC	VIM	2,0E-259	1,13	0,752	0,087
SMC/SC/EC	S100A10	2,4E-92	1,13	0,810	0,301
SMC/SC/EC	TPM2	4,3E-73	0,99	0,541	0,148
SMC/SC/EC	MYL6	1,6E-37	0,86	0,764	0,558
SMC/SC/EC	CSRP1	8,5E-293	0,85	0,430	0,022
SMC/SC/EC	PMP22	2,5E-55	0,79	0,430	0,107
SMC/SC/EC	ACTG2	0,0E+00	0,78	0,223	0,003
SMC/SC/EC	GADD45B	6,9E-14	0,77	0,566	0,413
SMC/SC/EC	ADIPOQ	0,0E+00	0,77	0,364	0,006
SMC/SC/EC	ENSGALG00000005209	2,2E-184	0,76	0,227	0,009
SMC/SC/EC	TIMP3	4,0E-120	0,75	0,521	0,087
SMC/SC/EC	ACTA1	5,5E-102	0,67	0,169	0,010
SMC/SC/EC	ACTB	1,6E-28	0,64	0,967	0,906
SMC/SC/EC	RGS4	0,0E+00	0,63	0,269	0,004
SMC/SC/EC	FN1	1,1E-98	0,62	0,376	0,052
SMC/SC/EC	RGS2	5,8E-48	0,60	0,355	0,084
SMC/SC/EC	MYH11	0,0E+00	0,60	0,236	0,001
SMC/SC/EC	FABP4	2,3E-115	0,59	0,140	0,005
SMC/SC/EC	HSPA2	1,6E-04	0,57	0,632	0,574
SMC/SC/EC	RAMP2	1,1E-176	0,56	0,256	0,013
SMC/SC/EC	MID1IP1	2,0E-26	0,54	0,471	0,210
SMC/SC/EC	RASD1	1,3E-72	0,54	0,285	0,040
SMC/SC/EC	ENSGALG00000042108	3,2E-220	0,53	0,136	0,001
SMC/SC/EC	KLF2	7,4E-14	0,53	0,459	0,259
SMC/SC/EC	CALM	6,3E-20	0,50	0,574	0,338
SMC/SC/EC	FKBP12	1,4E-12	0,49	0,579	0,409
SMC/SC/EC	CYR61	1,8E-17	0,47	0,273	0,095
SMC/SC/EC	MYLK	3,3E-293	0,46	0,207	0,003
SMC/SC/EC	VWF	9,1E-225	0,25	0,136	0,001

Abbreviations: pct, percent; KC, keratinocytes; FB, fibroblasts
ERY, erythrocytes; EC, endothelial cells; SC, Schwann cells; SMC, smooth muscle cells

Supplementary Table S2. Gene expression levels in KRT9L3-positive (differentiated, interscale epidermis) versus KRT14L1-positive (non-differentiated) KC

Gene	P-value (adjusted)	Average Log(e) FC	pct.1	pct.2
CBP63-K	2,44E-82	2,83	0,633	0,273
KRT9L3	0,00E+00	2,27	1,000	0,156
CBP62-K	3,68E-101	2,17	0,523	0,122
FABP5	2,82E-227	1,51	0,881	0,204
EDQL-EDC	7,13E-105	1,44	0,556	0,129
LGALS1	1,93E-184	1,30	0,998	0,938
CSTA	2,70E-190	1,25	0,770	0,159
DSP	3,81E-148	1,23	0,953	0,600
KRT78L2	1,20E-273	1,19	0,867	0,117
ELOVL4	0,00E+00	1,18	0,757	0,044
KRT78L3	0,00E+00	1,16	0,809	0,048
KRT9L4	2,50E-168	1,14	0,403	0,015
EDQM1-EDC	1,07E-129	1,12	0,387	0,030
ENSGALG00000043582	9,69E-185	0,98	0,529	0,041
ENSGALG00000005619	1,06E-124	0,91	0,944	0,447
ENSGALG00000007153	4,64E-136	0,90	0,899	0,419
DBI	6,38E-112	0,86	0,917	0,519
ENSGALG00000027207	1,09E-134	0,86	0,977	0,642
EDQM2-EDC	1,03E-117	0,84	0,351	0,026
ENSGALG00000045042	8,64E-242	0,84	0,633	0,042
NTM	1,14E-258	0,81	0,714	0,057
ENSGALG00000007127	3,75E-222	0,79	0,570	0,033
GLTP	3,38E-204	0,77	0,712	0,098
ENSGALG00000028520	9,97E-33	0,75	0,770	0,638
POF1B	3,10E-184	0,71	0,615	0,069
DYNLL1	1,59E-126	0,70	0,669	0,163
RALBP1	1,56E-69	0,65	0,707	0,319
ENSGALG00000025772	2,49E-194	0,65	0,466	0,018
ENSGALG00000027536	3,21E-92	0,63	0,836	0,404
ENSGALG00000045319	3,88E-83	0,63	0,419	0,079
ELOVL7	3,77E-165	0,63	0,588	0,074
ENSGALG00000029833	1,60E-160	0,62	0,615	0,086
FDPS	9,84E-132	0,62	0,399	0,031
CD9	4,00E-192	0,62	0,590	0,054
TMEM254	4,02E-112	0,61	0,390	0,041
ABHD12B	9,09E-150	0,60	0,525	0,062
GJA1	1,40E-92	0,59	0,682	0,224
CYSTM1	1,46E-156	0,55	0,450	0,032
SCCPDH	1,38E-118	0,54	0,532	0,092
JUP	7,49E-73	0,53	0,833	0,443
ND5	5,13E-23	0,51	0,890	0,780
ATP6	1,88E-24	0,51	0,955	0,937
P4HB	6,82E-80	0,50	0,691	0,257
CLDN1	7,81E-61	0,48	0,646	0,269
ENSGALG00000015140	3,12E-72	0,47	0,664	0,247
LDHB	1,98E-58	0,47	0,732	0,371
KCTD1	2,00E-72	0,47	0,709	0,290
CALD1	7,63E-43	0,46	0,685	0,358
ERP29	1,20E-121	0,46	0,523	0,081
ANXA2	3,16E-58	0,46	0,959	0,786
ENSGALG00000013742	4,28E-118	0,45	0,455	0,058
ENSGALG00000021451	6,42E-73	0,45	0,579	0,181
S100A6	4,07E-25	0,44	0,966	0,951
CKB	1,43E-77	0,44	0,480	0,114
EPPKL1	1,39E-122	0,44	0,293	0,009
ENSGALG00000045989	8,44E-62	0,43	0,489	0,146
SCP2	3,44E-48	0,42	0,493	0,178
ENSGALG00000011483	2,42E-107	0,42	0,441	0,062
ENSGALG00000046652	2,07E-85	0,41	0,225	0,010
ENSGALG00000027494	1,17E-153	0,41	0,399	0,019
DYNLL2	1,74E-67	0,41	0,455	0,114
SPTAN1	9,53E-87	0,41	0,453	0,087
AFDN	2,00E-79	0,40	0,477	0,108
COX3	3,68E-22	0,39	0,973	0,987
ENSGALG00000003521	7,62E-55	0,39	0,583	0,224
ZNF706	2,27E-32	0,38	0,725	0,450
MTDH	3,95E-58	0,38	0,525	0,174
ENSGALG00000006723	7,15E-100	0,38	0,342	0,033
DSG1	1,54E-89	0,37	0,480	0,093
EDKMb-EDC	9,36E-74	0,37	0,297	0,037
ACLY	2,97E-88	0,37	0,376	0,054
SLC25A17	4,41E-78	0,36	0,448	0,094
SPTBN1	9,36E-56	0,36	0,507	0,166
MID1IP1	2,23E-33	0,36	0,441	0,178
HSP90AA1	5,85E-33	0,36	0,849	0,612
DUSP14	1,34E-71	0,35	0,408	0,084
ENSGALG00000015271	9,74E-76	0,35	0,396	0,073
ARF6	8,95E-51	0,34	0,565	0,206
BZW1	5,21E-55	0,34	0,534	0,182
CALML3	2,46E-41	0,34	0,946	0,790
ENSGALG00000007519	3,12E-78	0,34	0,381	0,064
HACD2	4,79E-109	0,33	0,403	0,045
ACTR3	1,71E-49	0,33	0,541	0,202
RPL22L1	9,63E-21	0,33	0,795	0,632
CD44	1,62E-22	0,33	0,462	0,234
MANF	1,09E-41	0,33	0,507	0,199
ENSGALG00000045170	9,07E-78	0,33	0,200	0,008
EPCAM	3,20E-36	0,33	0,689	0,366
RPS10-NUDT3	6,17E-43	0,33	0,989	0,928
SH3BGRL3	2,51E-37	0,32	0,477	0,193

Supplementary Table S2 (continued). Gene expression levels in KRT9L3-positive (differentiated, interscale epidermis) versus KRT14L1-positive (non-differentiated) KC

Gene	P-value (adjusted)	Average Log(e) FC	pct.1	pct.2
MSMO1	8,01E-60	0,32	0,342	0,069
CDH1	2,85E-32	0,32	0,547	0,263
KIF21A	4,82E-69	0,32	0,342	0,057
PHLDA2	5,99E-23	0,31	0,775	0,505
HEXB	4,95E-57	0,31	0,428	0,114
DSTN	5,33E-33	0,31	0,930	0,739
ACTB	2,31E-17	0,31	0,966	0,909
ACAT2	4,75E-58	0,30	0,347	0,072
FDFT1	5,42E-69	0,30	0,239	0,022
ARL8B	7,30E-92	0,30	0,347	0,039
KRT9LC2	2,04E-02	0,30	0,142	0,075
NUS1	1,47E-60	0,29	0,410	0,098
ENSGALG00000032438	2,01E-31	0,29	0,545	0,260
ENSGALG00000011324	5,03E-45	0,29	0,446	0,144
ATP5D	1,23E-29	0,29	0,820	0,557
ASAH1	2,87E-31	0,29	0,565	0,267
RGS2	7,55E-41	0,29	0,245	0,048
ST13	8,14E-43	0,28	0,450	0,154
KLF6	8,65E-16	0,28	0,667	0,452
ENSGALG00000044962	4,48E-69	0,28	0,212	0,014
GPX1	3,19E-25	0,28	0,998	0,934
LAMTOR2	2,04E-46	0,28	0,437	0,138
TMEM41A	6,05E-55	0,28	0,378	0,090
HSP90B1	2,96E-32	0,28	0,450	0,182
ENSGALG00000007840	1,44E-78	0,28	0,239	0,016
TMEM79	4,74E-49	0,28	0,381	0,102
PSMA3	8,02E-37	0,27	0,523	0,218
MYCBP2	4,32E-42	0,27	0,414	0,130
NDUFA12	3,92E-26	0,27	0,536	0,272
GOLGA4	1,87E-51	0,26	0,309	0,062
FKBP12	5,79E-24	0,26	0,678	0,413
ENSGALG00000039191	2,47E-47	0,26	0,385	0,104
RTN4	2,68E-27	0,26	0,565	0,285
ABCA12	2,10E-111	0,26	0,284	0,011
CNN2	3,15E-30	0,26	0,426	0,172
HSD17B12	1,18E-46	0,26	0,349	0,088
ANK3	5,69E-48	0,25	0,338	0,080
HOPX	4,62E-36	0,25	0,349	0,107
KRT9LC1	5,32E-08	0,25	0,108	0,034
GCAT	8,85E-37	0,25	0,426	0,149
CAST	6,48E-41	0,25	0,403	0,127
EVPL	9,23E-74	0,25	0,279	0,030
ENSGALG00000008439	2,39E-98	0,24	0,230	0,005
ALDH3A1	4,18E-41	0,24	0,363	0,105
IL13RA2	9,83E-54	0,24	0,284	0,049
ENSGALG00000020485	7,76E-53	0,24	0,302	0,057
PPL	6,79E-72	0,24	0,286	0,033
PPDPF	6,92E-15	0,24	0,651	0,453
Galphai3	2,58E-26	0,24	0,509	0,242
NT5DC2	1,03E-34	0,23	0,313	0,091
KANK1	6,76E-46	0,23	0,302	0,066
FASN	9,40E-61	0,23	0,259	0,033
CAPNS2	1,22E-18	0,23	0,536	0,297
ENSGALG00000015345	5,89E-50	0,23	0,275	0,049
MVD	1,15E-64	0,23	0,248	0,027
ENSGALG00000015437	2,34E-26	0,23	0,360	0,139
ENSGALG00000046632	9,45E-22	0,23	0,164	0,038
SYNE2	7,84E-24	0,23	0,390	0,160
PRELID1	1,02E-14	0,23	0,757	0,556
HACD3	1,15E-40	0,22	0,322	0,083
PLS3	1,07E-22	0,22	0,374	0,156
TPM3	2,48E-14	0,22	0,489	0,292
ZDHHC6	1,84E-42	0,22	0,282	0,061
COX6A1	2,75E-12	0,22	0,813	0,684
IFRD1	1,80E-14	0,22	0,399	0,210
PGD	2,02E-24	0,22	0,392	0,162
HAGH	1,16E-45	0,22	0,257	0,047
PSMA7	2,61E-18	0,22	0,502	0,275
SDC1	2,52E-25	0,22	0,363	0,139
MDH2	2,55E-17	0,22	0,444	0,234
ANXA1	1,77E-05	0,22	0,455	0,318
PKP1	1,50E-49	0,21	0,277	0,051
SARS	2,27E-32	0,21	0,315	0,095
HOMER2	8,55E-42	0,21	0,277	0,060
CMPK1	1,10E-48	0,21	0,257	0,043
PEX11G	2,02E-56	0,21	0,250	0,034
PABPC1	3,74E-11	0,21	0,887	0,743
OAZ1	5,69E-14	0,21	0,802	0,604
MOGAT1	1,10E-88	0,21	0,194	0,002
RAB18	1,03E-20	0,20	0,349	0,147
BCAP29	3,17E-26	0,20	0,329	0,117
BARX2B	1,74E-32	0,20	0,203	0,039
NAP1L1	6,32E-17	0,20	0,520	0,287
IL36RN	7,33E-57	0,20	0,167	0,009
IL1RN	2,08E-58	0,20	0,218	0,022
CHD7	5,44E-25	0,20	0,261	0,080
SEPT5	2,26E-73	0,20	0,203	0,010
EPRS	1,33E-34	0,20	0,322	0,093
ENSGALG00000001000	1,53E-14	0,20	0,455	0,249
PHYH	8,19E-30	0,20	0,124	0,014

Supplementary Table S2 (continued). Gene expression levels in KRT9L3-positive (differentiated, interscale epidermis) versus KRT14L1-positive (non-differentiated) KC

Gene	P-value (adjusted)	Average Log(e) FC	pct.1	pct.2
PXMP4	1,67E-39	0,20	0,255	0,053
TRIP11	3,57E-38	0,20	0,259	0,056
ENSGALG00000013512	6,32E-15	0,19	0,615	0,382
RIOK1	6,31E-29	0,19	0,236	0,060
ELOVL6	4,73E-44	0,19	0,205	0,029
KTN1	5,46E-22	0,19	0,320	0,122
MAFB	1,15E-09	0,19	0,577	0,389
CLIC3	1,30E-23	0,19	0,315	0,114
LSS	3,04E-60	0,19	0,194	0,014
LANCL1	1,21E-23	0,19	0,309	0,110
EIF2B2	1,40E-34	0,19	0,270	0,067
ENSGALG00000011930	1,67E-62	0,19	0,155	0,004
GK5	2,82E-49	0,19	0,218	0,029
GAPDH	5,69E-09	0,19	0,948	0,922
OLAH	5,86E-81	0,19	0,187	0,004
NDUFA4	5,56E-10	0,19	0,604	0,422
HYPK	9,37E-12	0,19	0,399	0,226
ARPC3	5,51E-19	0,19	0,387	0,177
ENSGALG00000029002	2,56E-45	0,19	0,230	0,036
ENSGALG00000011687	3,40E-05	0,19	0,547	0,397
ESRP2	3,29E-22	0,19	0,349	0,138
LPCAT3	9,77E-40	0,18	0,236	0,045
ATP6V1G1	5,22E-15	0,18	0,527	0,303
YBX3	7,30E-12	0,18	0,489	0,298
PDLIM5	3,03E-54	0,18	0,205	0,021
CHL1	3,97E-24	0,18	0,291	0,098
C19orf24	3,32E-31	0,18	0,297	0,086
LGALSL	4,13E-20	0,18	0,329	0,132
LOR1-EDC	5,16E-38	0,18	0,104	0,004
KRT9L1	4,86E-54	0,18	0,153	0,008
ARPC4	3,24E-14	0,18	0,507	0,288
NEU2	5,98E-39	0,18	0,173	0,022
CHD4	2,99E-20	0,18	0,333	0,135
IDH1	1,21E-18	0,18	0,268	0,101
PSMD7	2,21E-14	0,18	0,365	0,183
IRAK2	3,50E-31	0,18	0,218	0,048
CLTB	5,24E-18	0,18	0,338	0,146
ORMDL2	6,86E-16	0,18	0,333	0,152
CYP51	5,43E-49	0,17	0,191	0,020
HSBP1L1	5,05E-32	0,17	0,236	0,055
PSMB1	1,25E-15	0,17	0,529	0,294
BDH1	3,10E-40	0,17	0,232	0,042
CHMP4B	7,34E-12	0,17	0,473	0,278
AACS	9,24E-47	0,17	0,189	0,021
cRac1A	1,92E-12	0,17	0,466	0,268
RCAN1	4,87E-33	0,17	0,194	0,035
PPA1	1,76E-20	0,17	0,297	0,112
DNAJA2	8,25E-18	0,17	0,313	0,130
EIF5B	9,83E-13	0,17	0,383	0,199
TSPO	8,14E-09	0,17	0,617	0,432
TPMT	4,93E-29	0,17	0,245	0,064
ENSGALG00000001986	5,53E-22	0,17	0,255	0,083
RAB10	5,00E-16	0,17	0,320	0,142
STUB1	3,32E-32	0,17	0,225	0,050
SLK	7,42E-15	0,17	0,349	0,163
PSMB2	1,33E-10	0,17	0,432	0,258
RPS27A	9,74E-04	0,17	0,986	0,992
ENSGALG00000029109	8,87E-32	0,17	0,214	0,045
GM2A	9,95E-24	0,17	0,248	0,075
TRIM35	5,44E-20	0,16	0,300	0,113
SFN	2,72E-32	0,16	0,198	0,038
GARS	1,38E-26	0,16	0,245	0,068
HMGCR	6,32E-31	0,16	0,212	0,045
HDLBP	5,77E-19	0,16	0,304	0,120
PARD3	5,28E-19	0,16	0,291	0,110
PSMA4	8,15E-11	0,16	0,421	0,244
PLK3	4,25E-41	0,16	0,203	0,030
LMO7	4,36E-21	0,16	0,223	0,067
ENSGALG00000006613	2,05E-46	0,16	0,196	0,024
NDUFA8	4,27E-13	0,16	0,414	0,223
SERPINB5	5,43E-26	0,16	0,245	0,069
RPIA	9,63E-31	0,16	0,225	0,051
RAB1A	2,82E-09	0,16	0,509	0,327
CAT	1,68E-20	0,16	0,214	0,064
DIAPH1	2,95E-17	0,16	0,297	0,121
TRIM29	7,54E-16	0,16	0,322	0,142
PDIA3	8,09E-13	0,16	0,394	0,207
ENSGALG00000028598	4,49E-49	0,16	0,126	0,004
RPL28	2,96E-08	0,16	0,980	0,988
EIF4G2	2,81E-06	0,16	0,509	0,354
TALDO1	4,72E-13	0,16	0,342	0,169
IL20RA	1,71E-40	0,15	0,189	0,026
LAMTOR5	8,30E-19	0,15	0,266	0,097
CSNK1A1	1,08E-13	0,15	0,336	0,159
HSDL1	9,93E-43	0,15	0,144	0,011
MEMO1	3,24E-32	0,15	0,203	0,039
CTNNB1	3,59E-06	0,15	0,428	0,283
ENSGALG00000015805	9,15E-17	0,15	0,270	0,105
B3GNT2	1,43E-20	0,15	0,227	0,070
EIF2B	3,14E-11	0,15	0,351	0,184

Supplementary Table S2 (continued). Gene expression levels in KRT9L3-positive (differentiated, interscale epidermis) versus KRT14L1-positive (non-differentiated) KC

Gene	P-value (adjusted)	Average Log(e) FC	pct.1	pct.2
WAPL	4,90E-17	0,15	0,236	0,084
DAAM1	3,91E-16	0,15	0,275	0,109
HSPBP1	1,20E-20	0,15	0,275	0,096
PDAP1	2,98E-17	0,15	0,275	0,106
SERPINE2	4,02E-16	0,15	0,218	0,076
ENSGALG00000043352	4,65E-48	0,15	0,164	0,013
CD109	6,96E-20	0,15	0,209	0,062
DYNLT3	1,17E-24	0,15	0,209	0,053
RPS24	1,44E-05	0,15	0,980	0,988
BAG3	4,80E-09	0,15	0,403	0,233
ENSGALG00000008105	4,59E-51	0,15	0,164	0,012
ARHGEF9	5,39E-39	0,15	0,173	0,022
PMVK	3,40E-13	0,15	0,241	0,099
ERP44	1,85E-25	0,14	0,196	0,046
PLIN2	3,95E-10	0,14	0,232	0,103
TAF10	3,70E-24	0,14	0,218	0,059
ENSGALG00000039500	3,27E-18	0,14	0,252	0,090
PANK1	1,08E-32	0,14	0,169	0,026
RPLP2	5,12E-04	0,14	1,000	0,997
CDKN1A	1,95E-09	0,14	0,295	0,150
FNIP2	1,62E-21	0,14	0,182	0,046
CCT5	1,30E-09	0,14	0,313	0,163
ABHD5	2,56E-17	0,14	0,187	0,056
PSMA6	5,49E-09	0,14	0,509	0,321
PCYOX1L	3,65E-22	0,14	0,209	0,058
DUSP4	7,83E-33	0,14	0,155	0,021
TXNDC17	1,07E-07	0,14	0,417	0,254
PSMC5	5,75E-09	0,14	0,385	0,221
SQLE	5,80E-46	0,14	0,144	0,009
INSIG1	2,97E-23	0,14	0,155	0,031
UBTD1	3,36E-18	0,14	0,212	0,067
ATP2A2	4,75E-11	0,14	0,241	0,105
ABCA3	1,20E-21	0,14	0,180	0,044
SERBP1	7,67E-06	0,14	0,759	0,614
LPAR2	4,00E-14	0,14	0,230	0,088
ENSGALG00000039156	2,43E-62	0,14	0,137	0,001
EDF1	8,29E-07	0,14	0,466	0,307
PDZK1IP1	2,46E-48	0,14	0,113	0,002
KLHL12	4,07E-10	0,14	0,264	0,125
PSAT1	2,46E-25	0,14	0,167	0,033
BCAS2	3,31E-11	0,13	0,277	0,129
SMDT1	1,49E-11	0,13	0,291	0,137
SREBF2	6,27E-15	0,13	0,227	0,084
VPS29	1,27E-13	0,13	0,230	0,090
RNF11	1,34E-17	0,13	0,212	0,068
DEGS1	6,97E-12	0,13	0,239	0,101
TUBB	1,99E-09	0,13	0,372	0,205
ENSGALG00000013232	7,11E-30	0,13	0,167	0,028
ATP6V0C	3,60E-04	0,13	0,523	0,379
WIPI1	4,70E-12	0,13	0,236	0,098
SBDS	3,51E-12	0,13	0,227	0,092
UQCRB	4,38E-08	0,13	0,367	0,209
ACAA2	3,54E-11	0,13	0,250	0,110
BLMH	1,63E-12	0,13	0,180	0,063
APMAP	3,00E-18	0,13	0,200	0,060
MRPL27	1,79E-15	0,13	0,216	0,076
RAB24	7,28E-25	0,13	0,173	0,036
RHOB	6,73E-13	0,13	0,270	0,115
SEPT8	4,40E-21	0,13	0,169	0,040
WDR53	4,76E-15	0,13	0,205	0,071
TOM1	4,08E-23	0,13	0,162	0,034
SCIN	3,68E-35	0,12	0,137	0,014
MAPKAPK3	3,76E-14	0,12	0,236	0,091
SMPD1	2,80E-13	0,12	0,234	0,092
ENSGALG00000004725	1,09E-08	0,12	0,255	0,124
PSMD1	2,70E-10	0,12	0,212	0,089
ENSGALG00000015702	9,52E-07	0,12	0,207	0,099
MICALL1	2,58E-11	0,12	0,218	0,089
ATP5H	1,14E-04	0,12	0,547	0,398
ENSGALG00000035774	2,83E-15	0,12	0,216	0,075
RAB11A	2,35E-08	0,12	0,261	0,129
CAPZB	7,26E-09	0,12	0,313	0,163
CGNL1	5,01E-20	0,12	0,164	0,039
ATP6V0B	1,10E-10	0,12	0,230	0,098
SPTY2D1	2,24E-08	0,12	0,207	0,093
DHCR7	8,08E-31	0,12	0,124	0,013
FRMD4B	4,36E-30	0,12	0,124	0,013
SYNGR2	8,83E-08	0,12	0,277	0,142
ENSGALG00000038876	5,65E-13	0,12	0,200	0,073
MACF1	3,20E-05	0,12	0,284	0,163
KLF2	4,40E-06	0,12	0,403	0,249
IQGAP1	2,08E-21	0,12	0,169	0,039
NDUFS3	1,27E-06	0,12	0,273	0,148
SCARB2	1,29E-09	0,12	0,232	0,104
ENSGALG00000032847	1,30E-03	0,12	0,381	0,258
C1orf21	8,40E-07	0,11	0,236	0,119
SS18	3,00E-08	0,11	0,230	0,108
ENSGALG00000044641	2,11E-08	0,11	0,212	0,095
ACAD9	1,64E-05	0,11	0,345	0,209
GLRX3	4,12E-10	0,11	0,223	0,096

Supplementary Table S2 (continued). Gene expression levels in KRT9L3-positive (differentiated, interscale epidermis) versus KRT14L1-positive (non-differentiated) KC

Gene	P-value (adjusted)	Average Log(e) FC	pct.1	pct.2
PGLS	7,31E-03	0,11	0,480	0,358
ENSGALG00000009325	3,97E-10	0,11	0,191	0,076
HYOU1	3,34E-19	0,11	0,169	0,043
ANXA4	6,78E-10	0,11	0,236	0,105
PTTG1IP	3,49E-09	0,11	0,205	0,088
THOC2	1,04E-12	0,11	0,178	0,060
DYNC1H1	2,81E-10	0,11	0,223	0,095
CACYBP	2,44E-11	0,11	0,216	0,087
KIF5B	1,54E-10	0,11	0,191	0,075
FLNB	2,01E-12	0,11	0,173	0,059
POLR2E	1,05E-07	0,11	0,282	0,146
ENSGALG00000021636	3,73E-10	0,11	0,187	0,073
CTSA	2,87E-06	0,11	0,187	0,087
GBF1	1,42E-15	0,11	0,173	0,051
BLCAP	1,07E-05	0,11	0,234	0,123
CLPX	1,47E-11	0,11	0,200	0,077
ENSGALG00000039257	7,63E-30	0,11	0,131	0,016
PSMD3	4,45E-08	0,11	0,248	0,121
ACAT1	1,37E-08	0,11	0,239	0,112
HDAC7	2,47E-16	0,11	0,158	0,042
ENSGALG00000045194	3,80E-45	0,11	0,104	0,001
RAB21	2,56E-11	0,11	0,200	0,077
UBXN4	8,33E-06	0,11	0,282	0,158
CNBP	4,76E-02	0,11	0,640	0,518
TMCO1	1,68E-08	0,11	0,266	0,130
AR	1,74E-10	0,11	0,187	0,072
BZW2	4,09E-06	0,11	0,257	0,137
AGPS	8,73E-21	0,11	0,135	0,026
CORO1C	7,46E-06	0,11	0,275	0,152
ENSGALG00000033993	2,24E-05	0,11	0,311	0,183
ERC1	1,34E-07	0,11	0,200	0,091
NADK	4,26E-16	0,11	0,146	0,037
MXD1	6,10E-17	0,11	0,140	0,033
AIG1	8,24E-14	0,11	0,171	0,054
EMC6	2,05E-05	0,11	0,227	0,121
MFSD2A	7,38E-06	0,11	0,191	0,093
UHMK1	1,70E-19	0,11	0,146	0,032
ENSGALG00000038671	9,87E-08	0,11	0,191	0,084
ACSL1	9,24E-09	0,11	0,178	0,072
HABP4	1,63E-23	0,11	0,135	0,023
RBX1	3,46E-04	0,11	0,291	0,176
HSPA4L	1,32E-08	0,11	0,218	0,098
ADK	2,89E-13	0,11	0,167	0,053
MED19	4,40E-15	0,11	0,137	0,035
CHP1	3,38E-09	0,10	0,198	0,083
SEPT7	3,54E-02	0,10	0,313	0,213
PSMC1	8,11E-06	0,10	0,241	0,127
EIF2S1	1,09E-05	0,10	0,268	0,147
FAM195B	9,16E-05	0,10	0,291	0,172
SCEL	1,08E-12	0,10	0,160	0,051
ARPC2	2,23E-03	0,10	0,414	0,283
NAA15	3,09E-13	0,10	0,140	0,039
GNL3	5,89E-06	0,10	0,218	0,110
MAP7	4,69E-14	0,10	0,153	0,044
RRS1	4,50E-06	0,10	0,277	0,151
ENSGALG00000037860	4,46E-13	0,10	0,155	0,047
RPS13	1,65E-03	0,10	0,982	0,974
COPE	1,23E-06	0,10	0,230	0,115
UPF3B	2,36E-07	0,10	0,169	0,071
TGS1	1,55E-13	0,10	0,135	0,036
UBE2V2	1,08E-07	0,10	0,187	0,081
KIF1B	1,06E-14	0,10	0,144	0,039
ZC3H15	4,63E-06	0,10	0,329	0,189
GATAD1	8,87E-18	0,10	0,142	0,032
DER	5,92E-10	0,10	0,173	0,065
GDI2	6,19E-06	0,10	0,275	0,150
ENSGALG00000009879	2,19E-12	0,10	0,169	0,056
PTPRF	4,55E-05	0,10	0,297	0,173
ACOT7	4,70E-10	0,10	0,173	0,065
ENSGALG00000038092	2,87E-09	0,10	0,185	0,074
ENSGALG00000043415	4,41E-25	0,10	0,122	0,016
DHCR24	7,57E-18	0,10	0,131	0,028
RASSF3	5,35E-16	0,10	0,140	0,034
SCNN1B	2,31E-08	0,10	0,216	0,097
SMARCA5	9,39E-06	0,10	0,223	0,114
FGFBP1	4,87E-09	0,10	0,164	0,063
JPT1	5,86E-06	0,10	0,212	0,106
SFT2D1	2,48E-10	0,10	0,176	0,065
WNT5A	2,11E-06	0,10	0,169	0,075
EVL	6,12E-14	0,10	0,140	0,038
NDUFV1	6,12E-04	0,10	0,358	0,232
RAB14	2,89E-07	0,10	0,214	0,101
MVK	5,73E-11	0,10	0,151	0,050
CLIP1	2,96E-08	0,10	0,158	0,061
SNX1	1,61E-10	0,10	0,146	0,048
ENSGALG00000021450	2,77E-21	0,10	0,122	0,020
CPM	2,52E-17	0,10	0,108	0,019
CCT2	1,34E-04	0,10	0,293	0,173
SDF2	1,04E-09	0,10	0,173	0,066
Mtch2	1,96E-05	0,10	0,261	0,143

Supplementary Table S2 (continued). Gene expression levels in KRT9L3-positive (differentiated, interscale epidermis) versus KRT14L1-positive (non-differentiated) KC

Gene	P-value (adjusted)	Average Log(e) FC	pct.1	pct.2
TTLL5	3,29E-19	0,10	0,122	0,022
CDC42BPB	7,51E-05	0,10	0,248	0,137
CTNND1	4,39E-04	0,10	0,291	0,175
SRP72	1,32E-07	0,10	0,191	0,084
NTMT1	3,25E-05	0,10	0,252	0,138
CLSTN1	2,24E-04	0,10	0,255	0,145
ENSGALG00000014846	2,96E-07	0,10	0,185	0,081
IDH2	6,97E-06	0,10	0,214	0,107
TCIM	5,25E-06	0,10	0,198	0,095
GNPAT	9,48E-20	0,10	0,104	0,015
PDCL3	1,24E-06	0,10	0,205	0,097
TEC	4,56E-32	0,10	0,104	0,007
ENSGALG00000006326	8,34E-03	0,10	0,703	0,549
ENSGALG00000012610	2,80E-05	0,10	0,171	0,080
EMC3	1,78E-08	0,10	0,142	0,051
APPL2	3,66E-24	0,10	0,119	0,016
CITED4	4,44E-17	0,10	0,128	0,028
ENSGALG00000027704	2,14E-12	0,09	0,131	0,036
NDUFB10	9,48E-03	0,09	0,471	0,339
GADD45A	3,65E-04	0,09	0,309	0,187
KPNA4	4,68E-04	0,09	0,273	0,161
INPP5A	2,01E-05	0,09	0,209	0,106
ENSGALG00000017136	9,31E-16	0,09	0,126	0,028
ENSGALG00000005956	1,99E-11	0,09	0,119	0,032
PSMD12	8,34E-04	0,09	0,286	0,173
DAAM2	3,44E-21	0,09	0,104	0,014
ERO1A	4,94E-18	0,09	0,126	0,025
ENSGALG00000016410	1,24E-04	0,09	0,354	0,219
TMF1	3,01E-07	0,09	0,164	0,068
SEC11C	4,19E-03	0,09	0,230	0,135
RPS19	1,26E-03	0,09	0,998	0,999
EPB41L1	5,18E-12	0,09	0,135	0,039
FAM83H	1,97E-09	0,09	0,160	0,059
PDCD6IP	5,70E-03	0,09	0,284	0,178
MARK1	6,41E-16	0,09	0,122	0,026
ENSGALG00000041471	2,65E-07	0,09	0,182	0,079
ACO1	2,99E-15	0,09	0,128	0,030
RAB11B	1,45E-11	0,09	0,153	0,049
CDKN1B	1,27E-03	0,09	0,239	0,138
ARHGDIA	8,07E-04	0,09	0,257	0,150
ACSBG2	4,46E-10	0,09	0,155	0,054
GRPEL1	8,01E-09	0,09	0,162	0,062
CIB1	6,34E-06	0,09	0,205	0,100
PGRMC1	4,68E-09	0,09	0,169	0,065
MFN1	1,92E-12	0,09	0,122	0,031
ENSGALG00000000433	1,83E-23	0,09	0,104	0,012
EI24	1,39E-06	0,09	0,180	0,081
PPFIBP2	9,74E-05	0,09	0,187	0,094
CUL3	4,68E-15	0,09	0,110	0,023
BACH1	7,76E-17	0,09	0,117	0,023
CS	1,29E-19	0,09	0,117	0,020
AVPI1	3,47E-05	0,09	0,216	0,112
TIMP3	7,72E-05	0,09	0,160	0,074
IDNK	1,11E-18	0,09	0,106	0,017
ENSGALG00000001099	5,12E-05	0,09	0,196	0,098
FAM210B	5,04E-15	0,09	0,122	0,028
BTG2	2,01E-03	0,09	0,225	0,128
PLEKHA5	3,94E-15	0,09	0,115	0,024
CDV3	1,68E-03	0,09	0,250	0,147
ENSGALG00000044628	5,60E-05	0,09	0,205	0,105
ACP6	1,58E-13	0,09	0,128	0,033
UNC45A	9,95E-05	0,09	0,153	0,071
ACAA1	4,02E-15	0,09	0,128	0,030
RBBP6	6,65E-05	0,09	0,218	0,114
PSMC6	2,15E-04	0,09	0,230	0,126
PSMG3	7,56E-10	0,09	0,135	0,043
ARID4B	1,82E-02	0,09	0,221	0,132
EIF3A	2,21E-07	0,09	0,171	0,071
ENSGALG00000009475	2,70E-08	0,09	0,153	0,058
Rchy1	3,21E-04	0,09	0,198	0,104
ACBD7	1,79E-14	0,09	0,113	0,024
VPS26B	3,12E-19	0,09	0,113	0,019
NPC2	6,49E-06	0,09	0,196	0,094
UCHL3	5,36E-04	0,09	0,218	0,120
ENSGALG00000006477	3,06E-03	0,09	0,230	0,134
FAM84A	4,67E-19	0,09	0,106	0,016
PSMD6	1,05E-03	0,09	0,205	0,112
TAX1BP1	2,78E-03	0,09	0,245	0,145
SURF4	1,13E-05	0,09	0,171	0,079
USP7	5,73E-07	0,09	0,167	0,070
ENSGALG00000031002	1,15E-10	0,09	0,106	0,027
GAPVD1	8,59E-07	0,09	0,176	0,076
ABHD1	1,25E-04	0,09	0,167	0,080
DCTN2	4,02E-05	0,09	0,196	0,098
UBAC1	6,71E-15	0,09	0,122	0,028
WSB2	1,80E-04	0,08	0,189	0,096
PSMC3	4,25E-02	0,08	0,232	0,144
UGP2	1,70E-03	0,08	0,198	0,108
NIPA1	2,30E-13	0,08	0,108	0,024
ECI2	2,64E-09	0,08	0,133	0,043

Supplementary Table S2 (continued). Gene expression levels in KRT9L3-positive (differentiated, interscale epidermis) versus KRT14L1-positive (non-differentiated) KC

Gene	P-value (adjusted)	Average Log(e) FC	pct.1	pct.2
PPIG	3,23E-02	0,08	0,351	0,239
AMOT	3,77E-10	0,08	0,124	0,037
ENSGALG00000013036	3,31E-07	0,08	0,144	0,055
PITPNB	3,21E-03	0,08	0,268	0,162
PDCD4	3,11E-02	0,08	0,227	0,139
ENSGALG00000039412	4,57E-16	0,08	0,113	0,022
MRPL28	4,24E-06	0,08	0,171	0,076
MRPS31	2,51E-06	0,08	0,173	0,077
MVB12A	2,43E-05	0,08	0,185	0,089
ENSGALG00000035236	3,12E-02	0,08	0,331	0,222
SNX3	1,01E-04	0,08	0,173	0,084
SRP14	1,51E-06	0,08	0,160	0,067
PREB	5,27E-08	0,08	0,140	0,051
PIGA	1,91E-14	0,08	0,117	0,026
Zfp36l1	6,81E-07	0,08	0,153	0,062
ENSGALG00000031296	5,58E-03	0,08	0,176	0,094
PSMD2	5,75E-05	0,08	0,178	0,086
PALMD	1,52E-05	0,08	0,142	0,060
TMEM223	2,57E-02	0,08	0,255	0,160
ANKRD12	5,38E-06	0,08	0,137	0,055
ACOX1	1,44E-11	0,08	0,108	0,027
CLINT1	1,22E-04	0,08	0,185	0,092
ABCD3	3,58E-05	0,08	0,164	0,075
F2RL1	4,21E-16	0,08	0,104	0,019
ERLIN1	3,26E-07	0,08	0,144	0,055
SRA1	6,47E-04	0,08	0,227	0,126
SMAP2	2,03E-07	0,08	0,131	0,047
CANX	1,91E-03	0,08	0,243	0,141
NPTN	1,87E-03	0,08	0,198	0,108
PEX3	1,44E-09	0,08	0,128	0,040
BPTF	4,46E-05	0,08	0,142	0,061
MMADHC	2,01E-05	0,08	0,146	0,063
PAFAH1B1	8,22E-03	0,08	0,264	0,162
SASH1	1,04E-08	0,08	0,124	0,040
TOP2B	1,19E-07	0,08	0,122	0,041
CDC5L	2,55E-04	0,08	0,173	0,086
GAB1	5,37E-10	0,08	0,113	0,031
SLC20A2	4,57E-07	0,08	0,126	0,045
EN1	5,52E-11	0,08	0,108	0,028
UBE2A	1,08E-05	0,08	0,153	0,066
ENSGALG00000032537	4,62E-03	0,08	0,230	0,134
ENSGALG00000014071	4,05E-09	0,08	0,115	0,034
VMP1	8,48E-06	0,08	0,144	0,060
MYO6	1,21E-10	0,08	0,106	0,027
MRPS25	6,47E-04	0,08	0,158	0,077
JARID2	2,96E-05	0,08	0,142	0,060
VPS4B	5,36E-04	0,08	0,149	0,070
DHDH	6,44E-06	0,08	0,137	0,055
ENSGALG00000036791	1,03E-09	0,08	0,117	0,034
ENSGALG00000006474	2,14E-02	0,08	0,221	0,132
NOTCH2	1,38E-06	0,08	0,133	0,051
USP8	2,46E-04	0,08	0,178	0,088
ENSGALG00000008128	6,67E-03	0,08	0,196	0,110
ENSGALG00000034704	6,30E-05	0,08	0,126	0,052
CCDC6	5,85E-06	0,08	0,151	0,063
DCTN6	1,99E-03	0,08	0,185	0,098
NCOA4	4,77E-04	0,08	0,149	0,070
NR4A2	9,45E-03	0,08	0,209	0,119
ENSGALG00000013615	7,34E-05	0,08	0,135	0,058
ENSGALG00000039842	2,41E-06	0,07	0,117	0,042
AIMP2	1,02E-04	0,07	0,142	0,063
SIAH2	1,66E-10	0,07	0,115	0,031
FURIN	1,97E-05	0,07	0,144	0,061
MRPL14	1,55E-02	0,07	0,200	0,115
ENSGALG00000028484	2,03E-02	0,07	0,200	0,116
PHPT1	2,30E-05	0,07	0,149	0,064
SMARCA2	1,44E-05	0,07	0,149	0,063
ENSGALG00000008797	1,08E-02	0,07	0,205	0,117
CTNNA1	3,22E-02	0,07	0,295	0,191
DESI1	5,59E-11	0,07	0,101	0,024
TEX264	7,55E-04	0,07	0,171	0,086
PTGES3	1,82E-02	0,07	0,266	0,165
KDM5B	5,22E-04	0,07	0,128	0,057
ITSN2	4,53E-07	0,07	0,110	0,036
ENSGALG00000001054	1,37E-02	0,07	0,167	0,090
SRP54	4,25E-07	0,07	0,122	0,043
STX7	1,79E-06	0,07	0,117	0,042
CARS	1,16E-05	0,07	0,124	0,048
NEMF	5,05E-05	0,07	0,124	0,050
H2AFY	1,52E-04	0,07	0,137	0,060
ABRACL	1,07E-08	0,07	0,110	0,033
PPP2R2D	1,28E-02	0,07	0,200	0,114
HOOK3	2,73E-09	0,07	0,110	0,031
TFG	1,08E-03	0,07	0,160	0,079
ECI1	1,89E-03	0,07	0,167	0,085
PRELID3A	3,91E-08	0,07	0,113	0,035
NUDC	9,86E-05	0,07	0,122	0,050
ENSGALG00000010878	1,66E-06	0,07	0,110	0,038
NT5C2	8,62E-06	0,07	0,122	0,046
SLTM	5,82E-05	0,07	0,151	0,067

Supplementary Table S2 (continued). Gene expression levels in KRT9L3-positive (differentiated, interscale epidermis) versus KRT14L1-positive (non-differentiated) KC

Gene	P-value (adjusted)	Average Log(e) FC	pct.1	pct.2
KCTD2	9,13E-07	0,07	0,117	0,041
NUDCD3	2,00E-05	0,07	0,128	0,051
MARCH5	1,88E-03	0,07	0,144	0,069
HSCB	7,26E-03	0,07	0,137	0,067
TRAPPC3	1,28E-06	0,07	0,115	0,040
P4HA1	4,86E-05	0,07	0,115	0,044
MRPS36	2,82E-06	0,07	0,115	0,041
VPS33B	2,04E-02	0,07	0,162	0,087
CLUH	1,61E-05	0,07	0,108	0,039
PGAP2	3,02E-04	0,07	0,128	0,055
BRWD3	2,53E-07	0,07	0,106	0,033
MTPN	1,28E-02	0,07	0,124	0,059
TBCA	3,23E-02	0,07	0,182	0,103
ENSGALG00000011198	1,00E-05	0,07	0,124	0,047
ADPRH	1,43E-02	0,07	0,171	0,093
MAST4	5,08E-03	0,07	0,108	0,047
NFE2L1	1,42E-02	0,07	0,173	0,094
TCERG1	1,87E-03	0,07	0,158	0,078
SNAP29	3,00E-04	0,06	0,119	0,050
SEPT6	1,92E-04	0,06	0,106	0,041
PWWP2B	4,96E-05	0,06	0,117	0,046
VAMP4	3,03E-03	0,06	0,135	0,064
ENSGALG00000044210	1,28E-02	0,06	0,131	0,064
CTPS1	2,12E-02	0,06	0,126	0,062
BCLAF3	2,23E-05	0,06	0,104	0,037
C1orf123	2,11E-02	0,06	0,124	0,060
CAMSAP2	2,25E-06	0,06	0,106	0,035
SLMAP	4,04E-03	0,06	0,164	0,084
KATNBL1	4,57E-03	0,06	0,110	0,048
DYNC1I2	6,66E-04	0,06	0,140	0,064
HNRNPA2B1	6,95E-05	0,06	0,106	0,039
JPT2	2,23E-02	0,06	0,146	0,076
DNAJC1	3,78E-04	0,06	0,101	0,039
SLC35B1	3,48E-03	0,06	0,140	0,067
BRWD1	1,14E-02	0,06	0,115	0,053
MMD	1,91E-04	0,06	0,115	0,046
ENSGALG00000034354	2,67E-03	0,06	0,126	0,058
DCTN3	4,19E-03	0,06	0,128	0,060
ENSGALG00000041760	1,08E-04	0,06	0,117	0,047
SMIM12	1,42E-02	0,06	0,126	0,061
DLGAP4	4,80E-02	0,06	0,182	0,104
LRRC59	9,24E-03	0,06	0,126	0,060
KRAS	3,55E-02	0,06	0,137	0,071
TAF13	1,01E-04	0,06	0,104	0,039
WDR47	3,21E-03	0,06	0,115	0,051
SPATA2L	5,02E-04	0,06	0,108	0,043
ADAM10	6,94E-03	0,06	0,108	0,047
HIPK2	4,26E-02	0,06	0,126	0,063
CTSB	3,80E-03	0,06	0,108	0,047
PNRC2	1,15E-02	0,06	0,117	0,054
COPB2	2,56E-02	0,06	0,119	0,057
PSMG2	2,58E-02	0,06	0,119	0,057
TPRKB	1,25E-02	0,06	0,113	0,051
LUC7L3	4,14E-02	0,06	0,146	0,077
PHF3	4,39E-02	0,06	0,137	0,071
TET2	2,32E-03	0,06	0,115	0,050
SRPRA	7,95E-04	0,06	0,104	0,041
NSD3	7,46E-03	0,05	0,108	0,047
SPRYD7	3,00E-02	0,05	0,110	0,051
DPP7	3,09E-02	0,05	0,106	0,048
ENSGALG00000040586	3,28E-02	0,04	0,104	0,047
SLC4A7	7,83E-03	-0,07	0,029	0,104
KLF11	2,12E-02	-0,08	0,043	0,119
SIVA1	2,28E-02	-0,08	0,041	0,116
ENSGALG00000042611	3,56E-03	-0,08	0,027	0,103
USE1	4,75E-03	-0,08	0,038	0,119
PSMD10	8,19E-03	-0,09	0,065	0,154
ENSGALG00000008518	2,06E-03	-0,09	0,034	0,116
ENSGALG00000034784	3,42E-02	-0,10	0,083	0,171
ENSGALG00000036498	2,08E-06	-0,10	0,009	0,102
ACTN1	1,23E-02	-0,10	0,081	0,173
FKBP5	7,27E-03	-0,10	0,045	0,126
ENSGALG00000043071	8,17E-03	-0,10	0,083	0,177
ENSGALG00000009562	4,44E-03	-0,10	0,056	0,144
LIFR	5,42E-05	-0,10	0,027	0,120
LGR4	2,10E-03	-0,11	0,063	0,157
ADSS	4,10E-05	-0,11	0,047	0,153
CD151	3,25E-04	-0,11	0,050	0,146
AHCY	2,78E-04	-0,11	0,061	0,162
AIRC	1,76E-04	-0,11	0,065	0,170
TRA2B	9,27E-04	-0,12	0,101	0,211
SUMO3	3,36E-02	-0,12	0,189	0,297
APOA1	2,69E-06	-0,12	0,014	0,110
RPS20	8,08E-04	-0,12	0,959	0,994
CTDSPL	7,67E-03	-0,12	0,153	0,263
ENSGALG00000002932	2,03E-02	-0,12	0,955	0,975
HNRNPD	5,16E-04	-0,12	0,095	0,203
cNFI-A	3,79E-03	-0,12	0,122	0,229
ENSGALG00000040297	3,41E-03	-0,12	0,133	0,243
IRF1	2,55E-02	-0,12	0,050	0,127

Supplementary Table S2 (continued). Gene expression levels in KRT9L3-positive (differentiated, interscale epidermis) versus KRT14L1-positive (non-differentiated) KC

Gene	P-value (adjusted)	Average Log(e) FC	pct.1	pct.2
GNAI2	4,23E-08	-0,13	0,016	0,129
APP	7,01E-04	-0,13	0,092	0,198
NDUFB7	3,58E-02	-0,13	0,239	0,345
SUMO1	7,41E-03	-0,13	0,140	0,243
TIMM10	3,63E-03	-0,13	0,119	0,224
MRPS12	8,63E-03	-0,13	0,180	0,289
RPL27A	4,94E-04	-0,13	0,955	0,991
MRPL51	7,22E-03	-0,13	0,128	0,231
REEP5	3,23E-04	-0,13	0,158	0,284
WNT6	4,97E-08	-0,13	0,011	0,121
MEF2A	2,65E-05	-0,13	0,063	0,175
COX19	3,15E-05	-0,13	0,081	0,198
COL5A1	2,01E-08	-0,13	0,016	0,133
COX14	1,13E-05	-0,13	0,095	0,221
CDK6	1,56E-08	-0,13	0,020	0,140
SNRPD3	1,65E-02	-0,13	0,313	0,421
SNRPD1	6,63E-03	-0,13	0,255	0,372
KRT15	6,18E-08	-0,13	0,018	0,131
PTGS2	2,70E-05	-0,14	0,052	0,162
TPT1	3,99E-03	-0,14	0,989	0,996
TLCD1	7,70E-05	-0,14	0,113	0,233
ENSGALG00000042629	1,21E-07	-0,14	0,059	0,191
ENSGALG00000007728	3,19E-06	-0,14	0,052	0,168
CSTB	3,03E-08	-0,14	0,038	0,165
CD47	3,61E-03	-0,14	0,137	0,245
C11orf96	6,91E-06	-0,15	0,011	0,102
NDUFB1	7,02E-04	-0,15	0,214	0,335
ATP5G1	3,33E-02	-0,15	0,565	0,634
ITGA3	5,22E-07	-0,15	0,063	0,191
ENSGALG00000036057	1,08E-05	-0,15	0,104	0,231
FMC1	9,69E-09	-0,15	0,047	0,183
ENSGALG00000000137	3,55E-02	-0,15	0,277	0,378
TNS1	2,13E-11	-0,15	0,011	0,148
ENSGALG00000033569	2,91E-07	-0,15	0,155	0,312
ATP1A1	6,33E-10	-0,16	0,038	0,180
COX4I1	4,58E-03	-0,16	0,550	0,640
MSN	3,22E-08	-0,16	0,077	0,219
ENSGALG00000041778	2,14E-13	-0,16	0,005	0,152
PHB2	9,60E-04	-0,17	0,243	0,361
NDUFAF8	5,18E-09	-0,17	0,079	0,228
cRPS11	3,35E-10	-0,17	0,993	0,996
PHYHIPL	6,44E-07	-0,17	0,117	0,257
ENSGALG00000037920	5,09E-10	-0,17	0,072	0,229
ENSGALG00000023819	1,63E-04	-0,17	0,054	0,156
FGFR3	1,31E-06	-0,17	0,135	0,280
RPS6	2,13E-08	-0,17	0,937	0,969
ENSGALG00000044383	1,51E-02	-0,17	0,338	0,449
POSTN	4,99E-05	-0,18	0,104	0,226
DNTTIP2	2,37E-08	-0,18	0,158	0,321
ENSGALG00000016471	1,29E-13	-0,18	0,029	0,196
COL17A1	1,62E-09	-0,18	0,029	0,163
PEBP1	5,21E-07	-0,18	0,191	0,342
EIF1	2,33E-04	-0,18	0,768	0,790
ATP5I	2,10E-03	-0,18	0,390	0,495
ITGB1	1,29E-13	-0,18	0,025	0,189
SQSTM1	2,49E-06	-0,18	0,182	0,333
CAVIN1	3,43E-10	-0,18	0,079	0,240
ENSGALG00000036073	3,29E-07	-0,18	0,009	0,110
ENSGALG00000007863	2,64E-05	-0,19	0,396	0,520
COL3A1	1,24E-08	-0,19	0,018	0,137
ENSGALG00000030902	1,71E-05	-0,19	0,214	0,361
CD99	4,85E-16	-0,19	0,020	0,199
DDX5	2,85E-04	-0,19	0,541	0,626
ENSGALG00000001475	1,64E-04	-0,19	0,054	0,154
ATP5J2	3,35E-05	-0,20	0,412	0,537
ENSGALG00000029172	6,70E-07	-0,20	0,297	0,447
CIRBP	1,42E-05	-0,20	0,441	0,556
KRT5L1	3,96E-07	-0,20	0,054	0,179
SEC61G	1,47E-10	-0,20	0,207	0,395
ENSGALG00000017380	2,26E-06	-0,20	0,365	0,500
GLUL	5,82E-16	-0,20	0,025	0,206
NSA2	8,71E-11	-0,21	0,198	0,389
RPL24	8,71E-13	-0,21	0,939	0,994
NDFIP1	2,43E-08	-0,21	0,259	0,423
SLC25A3	9,51E-09	-0,21	0,309	0,475
ROMO1	1,99E-07	-0,21	0,313	0,468
ENSGALG00000006899	8,60E-05	-0,21	0,525	0,603
IP6K2	2,15E-09	-0,21	0,140	0,302
ENSGALG00000007220	3,12E-06	-0,22	0,559	0,665
PDXK	5,77E-14	-0,22	0,079	0,268
RPS3A	1,99E-16	-0,22	0,975	0,998
ENSGALG00000035996	2,03E-09	-0,23	0,268	0,438
ENSGALG00000042011	2,40E-08	-0,23	0,471	0,610
RPL35	1,52E-15	-0,23	0,928	0,988
RPS8	1,33E-20	-0,23	0,984	0,999
RPL15	2,15E-19	-0,24	0,966	0,996
SVBP	1,38E-15	-0,24	0,142	0,359
ENSGALG00000028600	4,84E-14	-0,24	0,944	0,984
RPLP0	1,26E-16	-0,24	0,944	0,989
RPL7	1,33E-17	-0,24	0,926	0,988

Supplementary Table S2 (continued). Gene expression levels in KRT9L3-positive (differentiated, interscale epidermis) versus KRT14L1-positive (non-differentiated) KC

Gene	P-value (adjusted)	Average Log(e) FC	pct.1	pct.2
ENSGALG00000031593	1,88E-15	-0,24	0,079	0,280
MYL6	1,60E-08	-0,24	0,525	0,637
ITM2A	1,45E-08	-0,25	0,561	0,676
COL1A2	2,79E-16	-0,26	0,011	0,188
C12orf66	4,17E-22	-0,26	0,921	0,988
RPS14	1,07E-23	-0,27	0,966	0,997
ENSGALG00000037017	3,28E-14	-0,27	0,282	0,488
ENSGALG00000016691	1,38E-09	-0,27	0,604	0,700
ENSGALG00000002771	1,27E-20	-0,28	0,023	0,236
RPL7A	2,37E-23	-0,28	0,932	0,990
TSC22D3	6,65E-14	-0,28	0,164	0,369
SEPW1	7,27E-17	-0,28	0,360	0,570
H2AFV	9,96E-10	-0,28	0,419	0,571
RPL22	1,20E-21	-0,28	0,856	0,957
ENSGALG00000031853	2,45E-21	-0,29	0,191	0,456
ENSGALG00000026970	1,19E-19	-0,29	0,045	0,263
RPS15A	7,82E-26	-0,29	0,930	0,993
RPL31	1,57E-24	-0,29	0,892	0,980
FAM213A	2,39E-22	-0,30	0,050	0,286
RPL23A	4,71E-30	-0,30	0,975	0,996
COX7A2	7,20E-18	-0,30	0,295	0,523
ITM2B	4,84E-18	-0,30	0,216	0,449
EEF1B2	2,58E-25	-0,30	0,903	0,980
RPL8	2,52E-31	-0,31	0,950	0,998
TEKT5	1,26E-12	-0,31	0,227	0,418
ENSGALG00000008620	4,17E-30	-0,31	0,932	0,988
EIF4EBP1	2,96E-16	-0,31	0,455	0,628
RPL10A	7,07E-36	-0,32	0,957	0,995
RPL11	5,11E-25	-0,32	0,822	0,984
ENSGALG00000001250	3,98E-25	-0,32	0,151	0,429
HSPB1	4,03E-22	-0,32	0,072	0,316
RPL19	4,98E-31	-0,33	0,932	0,994
DUSP1	2,02E-08	-0,33	0,457	0,603
RPL3	1,36E-29	-0,33	0,874	0,978
ENSGALG00000043379	3,19E-34	-0,33	0,914	0,997
MT4	2,54E-04	-0,34	0,205	0,334
RPS15	9,21E-47	-0,35	1,000	1,000
RPL27	2,56E-34	-0,35	0,890	0,969
RPS16	4,48E-35	-0,36	0,905	0,983
MGP	2,50E-21	-0,37	0,023	0,241
S100A10	5,92E-21	-0,37	0,090	0,333
ENSGALG00000001634	6,93E-54	-0,37	0,993	1,000
ATIC	7,56E-32	-0,38	0,117	0,431
RPL26	2,30E-41	-0,39	0,944	0,996
RPL9	1,25E-40	-0,39	0,899	0,994
RPL4	6,89E-45	-0,39	0,935	0,991
RPLP1	2,05E-45	-0,39	0,998	0,999
Wpkci-8	2,88E-19	-0,41	0,547	0,679
ATP1B3	1,38E-37	-0,41	0,155	0,504
RPL13	3,55E-46	-0,42	0,865	0,989
UBC	6,62E-29	-0,42	0,736	0,845
ENSGALG00000002286	1,36E-34	-0,43	0,809	0,909
COL14A1	2,80E-35	-0,43	0,072	0,398
FAU	3,75E-59	-0,43	0,966	0,997
RPL21	2,35E-71	-0,44	0,986	1,000
SEPP1	1,05E-39	-0,44	0,086	0,434
ENSGALG00000004509	7,84E-29	-0,47	0,937	0,983
RPL38	8,39E-46	-0,47	0,723	0,927
DCN	2,88E-47	-0,48	0,036	0,416
UBA52	1,44E-54	-0,48	0,874	0,969
BSG	3,01E-45	-0,49	0,110	0,487
RPL34	3,80E-59	-0,50	0,872	0,992
RPSA	3,40E-72	-0,50	0,939	0,996
NPM1	6,61E-49	-0,51	0,437	0,756
SAT1	4,47E-32	-0,52	0,378	0,654
SPARC	1,18E-40	-0,53	0,108	0,462
RPS17	1,56E-74	-0,54	0,971	0,999
RPL32	1,25E-88	-0,57	0,984	0,998
RPL30	6,74E-81	-0,58	0,912	0,993
RPS3	1,18E-75	-0,59	0,847	0,986
DST	2,64E-47	-0,60	0,061	0,449
RPS25	3,23E-85	-0,60	0,881	0,993
RPL37A	1,89E-86	-0,63	0,953	0,996
RPS12	2,16E-83	-0,65	0,955	0,992
KRT14L2	5,52E-25	-0,66	0,065	0,329
RPS28	6,30E-93	-0,70	0,829	0,983
FOS	3,20E-32	-0,70	0,475	0,747
ENSGALG00000026383	1,10E-69	-0,71	0,176	0,658
RPS27	6,17E-93	-0,77	0,908	0,989
RPS23	2,08E-99	-0,79	0,755	0,966
RPS4	2,28E-114	-0,87	0,876	0,992
ENSGALG00000004952	8,85E-110	-0,90	0,777	0,982
ENSGALG00000023818	8,54E-80	-1,04	0,484	0,884
RPL36	6,39E-105	-1,07	0,676	0,941
RPL37	2,16E-115	-1,12	0,755	0,980
RPL29	8,20E-132	-1,14	0,707	0,985
RPS21	3,14E-109	-1,17	0,561	0,937
ENSGALG00000012229	7,37E-116	-1,43	0,505	0,904
KRT14L1	1,35E-245	-1,58	0,162	1,000

Abbreviations: FC, fold change; pct, percent; KC, keratinocytes

Supplementary Table S3. Gene expression levels in KRT9LC2-positive (differentiated, scale epidermis) versus KRT14L1-positive (non-differentiated) KC

Gene	P-value (adjusted)	Average Log(e) FC	pct.1	pct.2
KRT9LC2	0,00E+00	2,07	1,000	0,073
EDQL-EDC	4,74E-121	1,84	0,614	0,129
KRT9LC1	0,00E+00	1,76	0,902	0,032
ENSGALG00000045319	2,07E-159	1,49	0,604	0,079
DSP	2,94E-128	1,42	0,941	0,600
FABP5	1,33E-183	1,32	0,854	0,202
ENSGALG00000027207	1,15E-161	1,28	0,989	0,642
ENSGALG00000007153	7,29E-157	1,15	0,939	0,420
KRT78L2	1,12E-215	1,14	0,806	0,118
POF1B	2,27E-241	1,05	0,734	0,068
ENSGALG00000029833	1,18E-252	0,99	0,801	0,085
ENSGALG00000046632	3,97E-244	0,96	0,646	0,038
CSTA	1,73E-149	0,96	0,742	0,159
ENSGALG00000005619	7,94E-111	0,95	0,934	0,447
RALBP1	2,79E-114	0,94	0,827	0,319
ENSGALG00000028520	2,50E-60	0,91	0,859	0,638
ENSGALG00000027536	1,76E-133	0,89	0,904	0,404
DYNLL1	3,75E-166	0,87	0,779	0,162
KRT78L3	7,95E-216	0,87	0,638	0,049
LGALS1	1,85E-104	0,84	0,992	0,938
ENSGALG00000043582	4,54E-141	0,80	0,465	0,041
GLTP	1,16E-199	0,77	0,734	0,097
ELOVL4	9,41E-224	0,77	0,630	0,043
CYSTM1	2,40E-218	0,74	0,580	0,032
JUP	1,65E-96	0,73	0,880	0,443
DBI	1,47E-83	0,71	0,891	0,518
EDQM1-EDC	3,39E-32	0,69	0,184	0,030
KRT9L3	2,75E-49	0,65	0,487	0,159
ENSGALG00000015140	8,61E-94	0,65	0,739	0,247
RGS2	3,47E-114	0,64	0,434	0,048
LGALSL	2,38E-124	0,63	0,649	0,131
CLDN1	1,66E-85	0,59	0,739	0,268
ELOVL7	4,00E-145	0,59	0,574	0,075
ENSGALG00000036110	2,02E-206	0,58	0,484	0,014
EDMTF1-EDC	3,78E-41	0,57	0,117	0,005
EPPKL1	1,47E-191	0,56	0,428	0,009
GJA1	1,06E-76	0,56	0,665	0,224
NTM	1,24E-165	0,56	0,574	0,057
SLC25A17	1,23E-145	0,55	0,622	0,091
ENSGALG00000046652	3,82E-180	0,55	0,412	0,010
RPL22L1	1,19E-54	0,55	0,915	0,631
HOPX	1,19E-122	0,54	0,604	0,107
CBP53-S	1,45E-167	0,53	0,364	0,005
LMO7	1,40E-142	0,53	0,548	0,066
SPINK6	1,49E-52	0,53	0,152	0,007
DYNLL2	1,69E-95	0,51	0,556	0,115
RPS10-NUDT3	1,38E-70	0,51	0,995	0,928
MTDH	5,34E-68	0,49	0,566	0,173
CTNNB1	3,32E-68	0,49	0,723	0,281
GCAT	1,72E-95	0,47	0,620	0,148
EDQM2-EDC	1,42E-17	0,47	0,130	0,026
DSG1	1,22E-102	0,47	0,532	0,093
AFDN	5,65E-86	0,46	0,516	0,108
MT4	1,30E-18	0,46	0,561	0,333
ABHD12B	1,86E-112	0,44	0,473	0,062
CAST	6,68E-70	0,41	0,505	0,126
ANXA2	3,33E-40	0,41	0,947	0,786
ENSGALG00000025772	1,06E-143	0,40	0,386	0,018
ENSGALG00000013742	2,78E-79	0,39	0,386	0,058
ENSGALG00000015271	3,39E-84	0,38	0,439	0,073
CKB	6,33E-64	0,37	0,473	0,115
CBP54-S	6,14E-46	0,37	0,109	0,002
ND5	7,77E-07	0,36	0,848	0,780
ENSGALG00000010979	4,41E-89	0,36	0,449	0,073
ENSGALG00000011324	1,97E-54	0,36	0,492	0,143
PTTG1IP	1,66E-61	0,36	0,404	0,088
P4HB	3,88E-43	0,36	0,612	0,257
TMEM79	1,10E-71	0,35	0,465	0,101
EPCAM	1,51E-35	0,35	0,710	0,368
ASAH1	7,08E-38	0,35	0,609	0,266
SPTAN1	2,28E-61	0,34	0,407	0,088
NEU2	1,39E-108	0,34	0,335	0,022
CAPNS2	8,66E-32	0,34	0,614	0,299
IL36RN	5,48E-77	0,33	0,213	0,009
METAP2	3,84E-39	0,33	0,551	0,222
ATP6	4,20E-08	0,33	0,955	0,936
CD9	2,06E-87	0,32	0,402	0,055
RORA	9,48E-33	0,32	0,511	0,213
ATP5D	7,35E-26	0,31	0,809	0,557
PABPC1	1,69E-17	0,31	0,891	0,742
ENSGALG00000027083	6,04E-131	0,31	0,277	0,002
MAFB	4,77E-24	0,31	0,668	0,389
RPS24	8,05E-28	0,31	0,997	0,988
IL1RN	2,47E-94	0,31	0,303	0,022
ENSGALG00000028598	5,83E-93	0,30	0,215	0,004
CDK2AP1	1,60E-50	0,30	0,455	0,127
ENSGALG00000028451	4,48E-09	0,30	0,838	0,778
IFRD1	2,07E-19	0,30	0,436	0,211
MANF	4,73E-41	0,30	0,537	0,199
ENSGALG00000007840	6,39E-111	0,30	0,314	0,016
EVPL	4,50E-88	0,29	0,319	0,029
PKP1	2,85E-69	0,29	0,338	0,050
HSP90AA1	2,65E-20	0,29	0,816	0,611

Supplementary Table S3 (continued). Gene expression levels in KRT9LC2-positive (differentiated, scale epidermis) versus KRT14L1-positive (non-differentiated) KC

Gene	P-value (adjusted)	Average Log(e) FC	pct.1	pct.2
ENSGALG00000039191	4,38E-44	0,29	0,388	0,104
SPTBN1	6,65E-30	0,29	0,431	0,165
KCTD1	9,79E-24	0,28	0,564	0,291
SCP2	7,37E-33	0,28	0,465	0,179
TFPI2	3,77E-102	0,28	0,266	0,010
Galphai3	3,97E-26	0,28	0,516	0,241
KLF6	2,82E-08	0,28	0,620	0,452
ARF6	2,99E-36	0,28	0,540	0,206
ZNF706	1,19E-18	0,27	0,699	0,449
GNB2L1	4,68E-23	0,27	0,992	0,971
HSBP1L1	1,77E-67	0,27	0,348	0,054
C19orf24	8,98E-54	0,27	0,383	0,085
ADK	1,66E-66	0,26	0,340	0,052
ENSGALG00000024296	1,67E-53	0,26	0,152	0,007
ERP29	4,35E-51	0,26	0,364	0,080
RPS27A	3,89E-11	0,26	0,992	0,992
LAMTOR2	2,78E-37	0,26	0,426	0,139
ENSGALG00000020078	3,25E-17	0,26	0,904	0,793
TSPO	3,32E-19	0,26	0,691	0,432
ENSGALG00000032438	2,29E-24	0,26	0,537	0,261
EDKMb-EDC	5,52E-57	0,26	0,271	0,037
SCNN1B	8,05E-44	0,26	0,375	0,097
SEPT5	2,08E-111	0,26	0,282	0,009
SCIN	2,12E-94	0,25	0,266	0,013
TPMT	3,41E-50	0,25	0,319	0,062
ENSGALG00000011687	2,45E-12	0,25	0,612	0,397
CDKN1B	9,60E-31	0,25	0,396	0,138
SERPINB2	2,94E-27	0,25	0,293	0,087
CALML3	2,66E-20	0,25	0,928	0,790
LDHB	1,10E-15	0,25	0,604	0,371
SARS	1,20E-41	0,25	0,362	0,094
ENSGALG00000007519	3,28E-46	0,25	0,311	0,064
ENSGALG00000036774	6,79E-17	0,25	0,971	0,966
CALD1	1,07E-12	0,25	0,572	0,358
CDKN1A	1,79E-25	0,25	0,391	0,149
ENSGALG00000021636	4,40E-45	0,25	0,327	0,073
PSMA3	4,46E-24	0,25	0,484	0,218
ACAA2	9,08E-37	0,25	0,372	0,110
DSTN	1,25E-17	0,25	0,918	0,739
ENSGALG00000013512	6,59E-18	0,24	0,641	0,383
CNN2	5,15E-25	0,24	0,420	0,171
COX3	3,82E-09	0,24	0,981	0,987
ARL8B	1,33E-55	0,24	0,274	0,039
YBX3	1,12E-17	0,24	0,537	0,299
HAGH	2,63E-51	0,23	0,285	0,047
SYNE2	1,27E-18	0,23	0,375	0,159
CHMP4B	4,30E-20	0,23	0,543	0,279
TRIM29	8,36E-28	0,23	0,391	0,141
PGLS	3,71E-17	0,23	0,598	0,358
TMEM41A	4,76E-36	0,23	0,335	0,090
NFKBIA	2,64E-04	0,23	0,766	0,648
TIMP3	8,60E-34	0,23	0,293	0,074
ENSGALG00000027494	2,99E-58	0,23	0,215	0,019
ENSGALG00000043352	7,43E-87	0,23	0,253	0,013
SEC11C	3,40E-25	0,22	0,364	0,134
RPLP2	2,58E-13	0,22	0,995	0,997
SERBP1	1,91E-10	0,22	0,769	0,613
ESRP2	1,48E-23	0,22	0,367	0,138
HACD2	1,11E-52	0,22	0,282	0,044
DAAM2	1,52E-81	0,22	0,242	0,013
CSNK1A1	4,25E-28	0,22	0,423	0,159
PPFIBP2	1,54E-29	0,22	0,314	0,093
MYCBP2	1,78E-22	0,22	0,348	0,129
PPL	4,01E-49	0,22	0,239	0,033
ANK3	2,58E-34	0,22	0,306	0,078
PARD3	1,22E-24	0,22	0,324	0,109
PLK3	1,60E-65	0,22	0,271	0,030
LANCL1	2,62E-31	0,22	0,354	0,109
ENSGALG00000020485	1,77E-41	0,22	0,282	0,057
MID1IP1	1,01E-09	0,22	0,340	0,179
BAG3	8,10E-13	0,22	0,434	0,233
ATP6V1G1	4,86E-17	0,21	0,553	0,303
RAB18	2,40E-23	0,21	0,380	0,148
FNIP2	4,05E-37	0,21	0,239	0,045
STUB1	2,85E-37	0,21	0,250	0,050
CBP55-S	1,75E-69	0,21	0,146	0,000
ENSGALG00000045042	2,75E-32	0,21	0,215	0,042
PDZK1IP1	1,40E-83	0,21	0,186	0,002
ENSGALG00000038671	2,92E-28	0,21	0,290	0,083
EREG	8,50E-66	0,21	0,157	0,003
TXNDC17	2,44E-18	0,20	0,505	0,255
ENSGALG00000040260	6,35E-38	0,20	0,266	0,055
MACF1	2,52E-18	0,20	0,378	0,163
EIF3J	3,17E-14	0,20	0,420	0,214
PXMP4	1,35E-39	0,20	0,266	0,053
PEX11G	1,77E-52	0,20	0,253	0,034
ENSGALG00000013615	1,81E-35	0,20	0,263	0,057
Lpin2	1,86E-37	0,20	0,242	0,046
BOK	5,79E-26	0,20	0,303	0,094
CD2AP	8,97E-33	0,20	0,255	0,058
CDH1	1,60E-10	0,20	0,452	0,262
TPM3	2,75E-13	0,20	0,511	0,291
RND3	1,19E-12	0,20	0,404	0,208

Supplementary Table S3 (continued). Gene expression levels in KRT9LC2-positive (differentiated, scale epidermis) versus KRT14L1-positive (non-differentiated) KC

Gene	P-value (adjusted)	Average Log(e) FC	pct.1	pct.2
TMEM254	8,80E-28	0,20	0,202	0,042
HEXB	1,90E-21	0,19	0,319	0,115
RAB11A	7,37E-23	0,19	0,348	0,128
ERC1	6,45E-26	0,19	0,298	0,091
HSP90B1	4,58E-13	0,19	0,372	0,183
NUS1	1,44E-27	0,19	0,319	0,098
MAP7	2,59E-41	0,19	0,250	0,044
PCYOX1L	1,02E-33	0,19	0,261	0,058
GOLGA4	2,75E-22	0,19	0,226	0,062
UBTD1	4,15E-25	0,19	0,247	0,067
RIOK1	1,48E-22	0,18	0,226	0,061
PAK1	1,38E-34	0,18	0,242	0,049
WAPL	5,48E-22	0,18	0,266	0,083
ENSGALG00000042330	1,22E-33	0,18	0,239	0,049
ENSGALG00000011483	3,03E-31	0,18	0,258	0,061
TRIP11	1,28E-27	0,18	0,234	0,056
DLX5	5,97E-13	0,18	0,346	0,161
ENSGALG00000015805	3,39E-17	0,18	0,279	0,104
EPRS	5,52E-21	0,18	0,279	0,093
TUBB3	2,27E-34	0,18	0,218	0,040
RPL6	6,69E-13	0,18	0,992	0,988
PDCD4	1,32E-14	0,18	0,319	0,138
RPS19	3,55E-16	0,18	1,000	0,999
ZDHHC6	2,19E-29	0,17	0,250	0,060
ENSGALG00000029002	6,69E-34	0,17	0,207	0,036
TRIM35	3,84E-18	0,17	0,303	0,114
CITED4	2,27E-40	0,17	0,202	0,028
ADGRL2	4,84E-32	0,17	0,239	0,051
ENSGALG00000038876	2,22E-22	0,17	0,250	0,074
ENSGALG00000003521	3,85E-09	0,17	0,399	0,224
DNAJA2	5,95E-16	0,17	0,316	0,130
FDPS	6,04E-28	0,17	0,176	0,031
EGLN1	8,90E-14	0,17	0,338	0,153
DHDH	7,08E-31	0,17	0,245	0,055
SERPINE2	4,89E-19	0,17	0,242	0,077
cRac1A	3,26E-08	0,17	0,444	0,266
CBP52L-S	1,49E-48	0,17	0,114	0,002
KIF21A	6,89E-21	0,17	0,210	0,056
BCAS2	1,03E-12	0,17	0,293	0,128
OAZ1	3,56E-06	0,17	0,771	0,604
SH3BGRL3	1,93E-12	0,17	0,386	0,192
MLF2	5,73E-11	0,17	0,367	0,189
ENSGALG00000039470	6,40E-13	0,16	1,000	1,000
DIAPH1	2,35E-13	0,16	0,285	0,120
KANK1	1,72E-23	0,16	0,239	0,065
TAX1BP1	4,22E-14	0,16	0,330	0,145
ALDH3A1	1,06E-17	0,16	0,285	0,104
IDH2	1,33E-16	0,16	0,282	0,106
ACTR3	7,00E-09	0,16	0,370	0,203
ENSGALG00000008105	1,39E-54	0,16	0,176	0,011
CBP56-K	1,91E-48	0,16	0,125	0,004
APCDD1	1,12E-40	0,16	0,181	0,021
PDAP1	2,13E-16	0,16	0,282	0,106
ENSGALG00000034704	3,21E-24	0,16	0,213	0,051
ARPC2	2,06E-09	0,16	0,484	0,284
MAL2	3,33E-71	0,16	0,165	0,003
ACBD7	1,28E-41	0,16	0,191	0,023
Id3	1,21E-12	0,15	0,266	0,109
ERO1A	2,79E-37	0,15	0,186	0,025
EDF1	1,31E-07	0,15	0,487	0,306
RNF11	1,88E-20	0,15	0,234	0,069
PLEKHA5	4,98E-30	0,15	0,165	0,025
ENSGALG00000015345	4,00E-19	0,15	0,189	0,049
MSMO1	7,90E-18	0,15	0,223	0,069
TUBB	7,16E-14	0,15	0,420	0,206
ENSGALG00000036099	3,54E-06	0,15	0,955	0,945
ANKRD12	2,26E-16	0,15	0,189	0,054
FRMD4B	5,76E-44	0,15	0,160	0,013
EIF2B2	1,48E-19	0,15	0,226	0,067
HDLBP	6,57E-14	0,15	0,290	0,120
ENSGALG00000039412	1,84E-35	0,15	0,170	0,022
SLC48A1	7,13E-19	0,15	0,210	0,060
NAA15	1,23E-26	0,15	0,191	0,039
PHLDA2	9,29E-06	0,15	0,694	0,506
HOOK3	2,94E-27	0,15	0,176	0,031
KLF2	2,91E-03	0,15	0,378	0,251
BCAP29	2,92E-12	0,15	0,274	0,116
PSMD10	1,86E-09	0,14	0,309	0,154
EXOC6	7,93E-37	0,14	0,178	0,023
CAPZB	1,77E-09	0,14	0,324	0,162
CLIP1	7,51E-20	0,14	0,215	0,060
TCIM	6,87E-09	0,14	0,221	0,095
MXD1	5,79E-26	0,14	0,170	0,031
EIF5B	9,23E-06	0,14	0,340	0,198
CYB5R3	1,66E-17	0,14	0,223	0,070
DUSP4	1,42E-27	0,14	0,146	0,021
PMVK	1,12E-13	0,14	0,253	0,097
GATAD1	1,76E-29	0,14	0,186	0,033
GAPVD1	1,19E-16	0,14	0,231	0,076
CGNL1	3,93E-23	0,14	0,181	0,039
DYNC1H1	2,82E-10	0,14	0,229	0,095
PPP2R2D	1,38E-13	0,14	0,282	0,114
SCARB2	3,54E-10	0,14	0,242	0,104

Supplementary Table S3 (continued). Gene expression levels in KRT9LC2-positive (differentiated, scale epidermis) versus KRT14L1-positive (non-differentiated) KC

Gene	P-value (adjusted)	Average Log(e) FC	pct.1	pct.2
ABCA12	3,59E-45	0,14	0,157	0,012
RPS13	5,92E-06	0,14	0,973	0,974
ACBD3	1,92E-13	0,14	0,255	0,099
APPL2	2,19E-34	0,13	0,152	0,017
WNK2	8,39E-54	0,13	0,141	0,004
MAPKAPK3	4,94E-13	0,13	0,239	0,090
CRIP2	2,70E-10	0,13	0,287	0,132
SDC4	2,21E-03	0,13	0,537	0,389
ENSGALG00000011153	3,40E-28	0,13	0,168	0,027
PRELID1	1,62E-03	0,13	0,697	0,556
SLC38A2	2,90E-02	0,13	0,566	0,437
FOXO1	2,14E-10	0,13	0,239	0,101
ENSGALG00000039156	9,49E-57	0,13	0,128	0,001
RASSF3	1,46E-22	0,13	0,168	0,034
DBNDD2	9,27E-08	0,13	0,199	0,086
ACADL	1,10E-11	0,13	0,247	0,101
CPM	4,51E-23	0,13	0,128	0,019
USP8	3,21E-12	0,13	0,231	0,089
NOTCH2	7,55E-16	0,13	0,178	0,050
BLCAP	7,88E-09	0,13	0,263	0,122
RPL28	1,61E-03	0,13	1,000	0,988
UBE2V2	3,16E-11	0,13	0,213	0,081
SMDT1	6,93E-08	0,13	0,274	0,136
B3GNT2	4,63E-14	0,13	0,210	0,070
UBE2H	1,18E-05	0,13	0,316	0,177
PTRH2	6,13E-07	0,13	0,338	0,187
FOXN1	1,96E-50	0,13	0,144	0,006
GM2A	4,49E-13	0,13	0,213	0,075
ENSGALG00000001986	9,97E-11	0,13	0,213	0,083
VPS29	5,30E-11	0,13	0,226	0,090
SDCBP	2,13E-06	0,13	0,362	0,208
PDCD6IP	2,59E-06	0,13	0,322	0,179
ARPC3	2,50E-07	0,13	0,330	0,178
ENSGALG00000006613	2,08E-26	0,13	0,152	0,023
ATP6V0C	3,67E-03	0,13	0,521	0,378
ENSGALG00000044641	4,42E-08	0,13	0,218	0,096
ENSGALG00000031538	2,25E-27	0,13	0,146	0,021
PSMC5	9,91E-07	0,13	0,383	0,221
SPTY2D1	8,74E-11	0,13	0,229	0,092
MEMO1	5,82E-22	0,13	0,178	0,039
ZFYVE21	1,24E-08	0,13	0,234	0,104
CMPK1	3,23E-20	0,12	0,181	0,043
RAB24	1,49E-21	0,12	0,170	0,036
ENSGALG00000045796	3,19E-50	0,12	0,112	0,001
BNIP3	6,33E-05	0,12	0,343	0,205
RAB21	2,05E-12	0,12	0,213	0,077
ENSGALG00000017186	2,66E-02	0,12	0,269	0,167
ABCA3	1,45E-17	0,12	0,173	0,044
SDC1	4,99E-06	0,12	0,266	0,139
EMP1	4,35E-02	0,12	0,346	0,235
IRAK2	2,31E-16	0,12	0,178	0,049
N4BP3	7,49E-28	0,12	0,149	0,021
CCDC6	1,50E-12	0,12	0,191	0,064
SLK	2,73E-05	0,12	0,295	0,163
SLC20A2	4,41E-17	0,12	0,173	0,045
CLDND1	7,99E-16	0,12	0,168	0,045
AREG	2,43E-22	0,12	0,144	0,025
ENSGALG00000016410	5,91E-06	0,12	0,372	0,218
PLS3	3,68E-05	0,12	0,282	0,156
ENSGALG00000007127	5,78E-15	0,12	0,141	0,034
PANK1	1,06E-21	0,12	0,144	0,026
ENSGALG00000010842	3,21E-09	0,12	0,202	0,081
HSDL1	9,55E-33	0,12	0,125	0,011
SYNGR2	1,02E-04	0,12	0,261	0,142
PEX16	2,04E-09	0,12	0,199	0,078
SEPT7	8,67E-04	0,12	0,340	0,212
CHL1	6,97E-08	0,12	0,221	0,097
CEP350	6,44E-25	0,12	0,138	0,020
ENSGALG00000029109	3,68E-16	0,11	0,168	0,044
ENSGALG00000011930	7,96E-42	0,11	0,114	0,004
MINDY1	5,30E-08	0,11	0,229	0,102
SRA1	3,51E-06	0,11	0,253	0,128
EEA1	2,48E-16	0,11	0,133	0,028
DAAM1	4,26E-03	0,11	0,199	0,109
ENSGALG00000003172	8,90E-43	0,11	0,128	0,006
WDR53	4,11E-11	0,11	0,194	0,070
CLIC3	3,56E-06	0,11	0,234	0,115
Zfp36l1	4,71E-12	0,11	0,184	0,061
MARK1	8,88E-24	0,11	0,152	0,026
EIF2S1	1,18E-07	0,11	0,293	0,146
VAPB	3,15E-06	0,11	0,301	0,160
GADD45A	2,87E-05	0,11	0,327	0,188
ELOVL6	3,20E-20	0,11	0,146	0,028
ENSGALG00000039958	8,92E-06	0,11	0,250	0,128
RAB11FIP4	3,18E-34	0,11	0,125	0,010
GNG10	3,08E-34	0,11	0,112	0,007
MICALL1	4,24E-07	0,11	0,202	0,089
EGR1	6,32E-11	0,11	0,197	0,071
STX7	2,63E-15	0,11	0,160	0,042
USP7	7,16E-12	0,11	0,197	0,069
SFN	1,68E-18	0,11	0,162	0,037
TALDO1	8,65E-05	0,11	0,295	0,169
ORMDL2	5,75E-06	0,11	0,285	0,152

Supplementary Table S3 (continued). Gene expression levels in KRT9LC2-positive (differentiated, scale epidermis) versus KRT14L1-positive (non-differentiated) KC

Gene	P-value (adjusted)	Average Log(e) FC	pct.1	pct.2
ENSGALG00000009879	2,91E-11	0,11	0,170	0,056
TOM1	1,19E-16	0,11	0,149	0,035
SFXN1	1,56E-07	0,11	0,231	0,106
CCPG1	2,44E-09	0,11	0,202	0,080
ARHGEF9	4,13E-23	0,11	0,138	0,022
C1orf21	5,37E-05	0,11	0,231	0,119
ENSGALG00000032444	1,73E-13	0,11	0,154	0,043
Ufd1l	3,84E-07	0,11	0,247	0,118
KIF5B	1,72E-09	0,11	0,194	0,074
TMEM250	5,74E-09	0,11	0,213	0,088
ENSGALG00000044628	1,82E-06	0,11	0,221	0,104
UBE2D3	3,39E-03	0,11	0,370	0,240
PWWP2B	6,76E-14	0,11	0,160	0,044
ENSGALG00000028135	1,52E-13	0,11	0,152	0,041
RCAN1	1,77E-11	0,11	0,130	0,035
ENSGALG00000029831	3,91E-04	0,11	0,263	0,148
TFDP2	1,36E-09	0,11	0,218	0,089
PAIP2	4,83E-05	0,11	0,316	0,182
ENSGALG00000034218	8,62E-06	0,11	0,287	0,154
SCCPDH	3,60E-07	0,11	0,207	0,092
ENSGALG00000012610	3,20E-08	0,11	0,194	0,079
LPCAT3	3,06E-14	0,11	0,162	0,045
DEGS1	1,74E-07	0,11	0,223	0,101
PLIN2	3,30E-05	0,11	0,210	0,103
HSPBP1	1,50E-08	0,11	0,223	0,096
HNRNPH3	1,20E-02	0,11	0,383	0,258
ERP44	3,34E-14	0,11	0,165	0,046
ZNF750	5,68E-27	0,10	0,130	0,016
Id1	1,83E-02	0,10	0,356	0,231
PELI1	5,69E-07	0,10	0,194	0,084
PIP4P2	2,19E-19	0,10	0,136	0,025
ENSGALG00000023787	9,01E-42	0,10	0,104	0,002
XPO6	6,14E-11	0,10	0,146	0,044
HABP4	1,21E-18	0,10	0,128	0,023
BZW1	1,27E-04	0,10	0,314	0,182
ENSGALG00000004725	1,44E-05	0,10	0,245	0,125
PDLIM5	1,28E-20	0,10	0,125	0,020
MED9	2,86E-03	0,10	0,255	0,149
ASPG	8,44E-10	0,10	0,104	0,026
ABI1	3,16E-05	0,10	0,226	0,114
ZCCHC11	1,34E-10	0,10	0,149	0,046
BPTF	4,25E-08	0,10	0,165	0,062
INPP5A	5,57E-05	0,10	0,213	0,106
MAP4K4	3,62E-19	0,10	0,109	0,016
RAB1A	3,70E-03	0,10	0,476	0,327
CTNND1	1,13E-02	0,10	0,282	0,174
ENSGALG00000001183	5,81E-08	0,10	0,168	0,064
ACLY	2,31E-10	0,10	0,162	0,054
SNX1	8,19E-11	0,10	0,154	0,048
TMCO1	1,17E-04	0,10	0,245	0,130
PIK3CB	4,14E-14	0,10	0,120	0,026
ENSGALG00000034509	7,77E-04	0,10	0,247	0,137
ENSGALG00000039500	1,41E-06	0,10	0,202	0,091
MITF	4,13E-13	0,10	0,141	0,037
CBP15-K	9,74E-35	0,10	0,106	0,005
DESI1	9,70E-17	0,10	0,125	0,024
ENSGALG00000006723	1,27E-10	0,10	0,122	0,033
AKAP9	1,03E-07	0,10	0,207	0,088
SBDS	7,19E-05	0,10	0,191	0,092
SERPINB5	1,29E-06	0,10	0,168	0,068
ABCD3	4,60E-09	0,10	0,194	0,075
UCHL3	1,30E-05	0,10	0,239	0,121
HYOU1	1,73E-11	0,10	0,146	0,043
UBE2J1	4,52E-05	0,10	0,189	0,089
NUB1	7,06E-09	0,10	0,152	0,052
KLHL18	7,62E-19	0,10	0,122	0,021
ENSGALG00000037316	4,19E-25	0,10	0,104	0,010
TMEM9B	1,13E-04	0,10	0,242	0,128
DR1	1,18E-07	0,10	0,162	0,062
SRSF5	5,05E-06	0,09	0,197	0,089
MED19	1,32E-13	0,09	0,138	0,035
BTBD1	1,24E-03	0,09	0,255	0,144
ENSGALG00000039257	1,53E-20	0,09	0,112	0,016
ATF4	4,89E-02	0,09	0,646	0,498
ENSGALG00000035774	2,20E-07	0,09	0,184	0,075
PSMD7	4,40E-03	0,09	0,298	0,182
CSNK2A2	1,76E-14	0,09	0,120	0,025
MRPL1	1,87E-07	0,09	0,176	0,070
EIF3A	2,74E-07	0,09	0,176	0,070
AVPI1	1,27E-05	0,09	0,229	0,113
CHMP4C	8,19E-26	0,09	0,104	0,009
IDH1	1,72E-04	0,09	0,202	0,101
MAFK	1,85E-02	0,09	0,279	0,172
AMOT	3,58E-12	0,09	0,136	0,036
EVL	2,63E-08	0,09	0,122	0,038
ARPC4	6,65E-03	0,09	0,431	0,288
EIF2B	2,04E-03	0,09	0,306	0,183
CLTC	9,19E-04	0,09	0,186	0,094
cRac1B	8,63E-14	0,09	0,114	0,024
BNIP3L	1,24E-06	0,09	0,152	0,058
STMP1	7,21E-03	0,09	0,266	0,159
SRP72	6,35E-06	0,09	0,189	0,084
PLEKHA7	1,31E-08	0,09	0,120	0,035

Supplementary Table S3 (continued). Gene expression levels in KRT9LC2-positive (differentiated, scale epidermis) versus KRT14L1-positive (non-differentiated) KC

Gene	P-value (adjusted)	Average Log(e) FC	pct.1	pct.2
RAB11B	6,37E-09	0,09	0,149	0,050
TAF10	1,50E-07	0,09	0,157	0,058
LSS	3,48E-18	0,09	0,101	0,014
CCT5	1,01E-02	0,09	0,271	0,163
PDE6D	1,24E-07	0,09	0,149	0,054
FAM160A1	2,39E-11	0,09	0,109	0,025
CHMP5	3,03E-03	0,09	0,189	0,099
COMMD2	2,25E-04	0,09	0,247	0,133
HDAC7	2,15E-10	0,09	0,141	0,042
LPAR2	6,97E-05	0,09	0,189	0,089
PIGA	1,57E-12	0,09	0,114	0,026
PRELID3A	3,17E-10	0,09	0,125	0,035
CLTB	1,16E-02	0,09	0,247	0,145
SMPD1	1,26E-04	0,09	0,189	0,090
RPIA	6,94E-09	0,09	0,152	0,051
ATP6V0B	1,87E-03	0,09	0,191	0,099
DER	1,87E-06	0,09	0,160	0,064
ENSGALG00000033852	1,16E-07	0,09	0,133	0,045
DUSP14	2,44E-04	0,09	0,178	0,085
FAM83H	9,95E-07	0,09	0,152	0,058
VSNL1	7,81E-07	0,09	0,138	0,050
HYPK	3,80E-02	0,09	0,343	0,226
ENSGALG00000000433	8,11E-21	0,08	0,101	0,012
CUL3	1,37E-11	0,08	0,104	0,023
ZNF330	2,21E-06	0,08	0,146	0,056
GAB1	1,51E-10	0,08	0,120	0,031
KCTD7	1,37E-04	0,08	0,168	0,076
RB1CC1	7,80E-12	0,08	0,117	0,028
ENSGALG00000038721	2,90E-03	0,08	0,231	0,128
MARCH5	3,59E-05	0,08	0,160	0,069
ENSGALG00000028636	5,26E-03	0,08	0,269	0,159
AIG1	7,66E-07	0,08	0,146	0,054
POP5	3,97E-07	0,08	0,122	0,040
HIP1R	3,22E-14	0,08	0,101	0,018
ENSGALG00000044666	7,68E-04	0,08	0,176	0,086
MFN1	1,00E-10	0,08	0,120	0,031
PSMD4	3,89E-02	0,08	0,242	0,146
IDNK	2,04E-15	0,08	0,101	0,017
MAST4	6,58E-07	0,08	0,133	0,047
ASH1L	6,27E-04	0,08	0,157	0,072
UBAC1	1,24E-11	0,08	0,114	0,027
GOLGA1	9,49E-12	0,08	0,106	0,023
SMARCA5	1,32E-03	0,08	0,215	0,114
CACYBP	1,12E-03	0,08	0,176	0,086
ENSGALG00000038092	2,98E-05	0,08	0,168	0,073
FBXO22	4,00E-05	0,08	0,136	0,054
ENSGALG00000006022	3,73E-02	0,08	0,354	0,233
ENSGALG00000001099	2,13E-03	0,08	0,189	0,097
IGF1R	3,53E-03	0,08	0,157	0,076
TMEM183A	1,24E-04	0,08	0,146	0,062
ERLIN1	1,78E-05	0,08	0,141	0,056
UBE2K	1,39E-02	0,08	0,176	0,093
JARID2	7,74E-04	0,08	0,136	0,058
CDC5L	2,35E-03	0,08	0,173	0,086
UBN2	7,64E-13	0,08	0,104	0,021
ACAT1	3,52E-02	0,08	0,199	0,112
ENSGALG00000041988	1,86E-02	0,08	0,258	0,153
ENSGALG00000002789	5,99E-10	0,08	0,109	0,027
TUBA1A	4,58E-04	0,08	0,721	0,558
KIF13A	2,44E-08	0,08	0,109	0,031
ENSGALG00000006474	3,37E-02	0,08	0,226	0,132
NPTN	6,92E-03	0,08	0,199	0,107
SLC35A4	1,00E-03	0,08	0,178	0,087
DYNC1I2	2,43E-05	0,08	0,154	0,064
PPP4R2	6,97E-06	0,08	0,109	0,036
HSPA4L	1,18E-03	0,07	0,194	0,098
ENSGALG00000042101	3,94E-07	0,07	0,109	0,033
CHMP2B	6,75E-07	0,07	0,104	0,031
PAIP1	2,75E-04	0,07	0,130	0,054
LACTB	1,63E-06	0,07	0,101	0,031
CBFB	7,28E-03	0,07	0,130	0,060
MRPL28	2,37E-03	0,07	0,160	0,077
WDR47	1,06E-04	0,07	0,128	0,051
VPS4B	1,92E-04	0,07	0,157	0,070
MSX2	1,35E-03	0,07	0,130	0,056
ECI2	2,79E-05	0,07	0,120	0,044
AP5S1	6,37E-03	0,07	0,136	0,063
ENSGALG00000025937	6,38E-04	0,07	0,133	0,057
MRPS36	2,65E-05	0,07	0,114	0,041
TOP2B	2,71E-05	0,07	0,114	0,041
ACP6	1,77E-07	0,07	0,109	0,032
SCEL	5,40E-05	0,07	0,130	0,051
RALGAPA1	2,93E-04	0,07	0,106	0,039
ACAA1	1,89E-07	0,07	0,104	0,030
SDF2	2,58E-03	0,07	0,144	0,066
SEPT8	1,37E-05	0,07	0,114	0,040
MMADHC	4,42E-03	0,07	0,138	0,064
ENSGALG00000005956	6,82E-07	0,07	0,106	0,032
PIGY	2,71E-02	0,07	0,176	0,094
SAP30	2,99E-04	0,07	0,122	0,049
COX7A2L	1,97E-02	0,07	0,189	0,102
SMAP2	5,23E-04	0,07	0,117	0,047
UNC5B	8,44E-04	0,07	0,109	0,043

Supplementary Table S3 (continued). Gene expression levels in KRT9LC2-positive (differentiated, scale epidermis) versus KRT14L1-positive (non-differentiated) KC

Gene	P-value (adjusted)	Average Log(e) FC	pct.1	pct.2
NSD3	3,16E-05	0,07	0,125	0,047
CARS	2,19E-03	0,07	0,114	0,047
UBE2A	7,30E-04	0,07	0,149	0,067
EMC3	1,58E-04	0,07	0,128	0,051
FASN	7,45E-07	0,07	0,109	0,034
MIA3	6,57E-03	0,07	0,149	0,071
ACSL1	4,17E-02	0,07	0,144	0,072
ENSGALG00000040586	6,84E-06	0,07	0,128	0,046
GIGYF2	2,96E-02	0,07	0,122	0,057
MPZL3	3,61E-02	0,07	0,157	0,081
NDEL1	1,95E-03	0,07	0,122	0,052
USP6NL	2,39E-05	0,07	0,101	0,033
ENSGALG00000027704	4,73E-05	0,07	0,104	0,035
VAMP4	9,52E-03	0,07	0,136	0,063
PLXNB2	2,87E-06	0,07	0,106	0,034
PER2	2,18E-02	0,06	0,125	0,058
SFT2D1	2,26E-02	0,06	0,136	0,065
ZFC3H1	2,08E-03	0,06	0,128	0,055
FAM207A	3,22E-02	0,06	0,130	0,062
MRPS18C	1,46E-02	0,06	0,130	0,061
EMC7	1,49E-02	0,06	0,130	0,061
ENSGALG00000037860	1,37E-02	0,06	0,109	0,047
ENSGALG00000036791	7,33E-06	0,06	0,104	0,033
ENSGALG00000041760	2,37E-04	0,06	0,120	0,047
ENSGALG00000034354	1,38E-02	0,06	0,125	0,057
FURIN	2,29E-02	0,06	0,130	0,062
DNAJC3	1,10E-02	0,06	0,117	0,051
MRPL27	2,85E-02	0,06	0,149	0,075
RC3H1	6,70E-03	0,06	0,117	0,051
ENSGALG00000041471	4,97E-02	0,06	0,154	0,079
LIN7C	1,98E-02	0,06	0,138	0,066
FGFR1OP2	2,19E-04	0,06	0,109	0,040
ENSGALG00000010209	5,25E-03	0,06	0,117	0,050
TET2	2,46E-04	0,06	0,125	0,050
PRRG4	2,82E-02	0,06	0,120	0,055
IQGAP1	2,58E-04	0,06	0,109	0,040
LAMTOR3	9,61E-03	0,06	0,120	0,053
SMARCA2	3,89E-02	0,06	0,133	0,064
EP300	4,68E-02	0,06	0,120	0,056
PEX3	1,55E-03	0,06	0,104	0,040
GPATCH3	2,76E-02	0,06	0,109	0,048
ENSGALG00000001317	1,70E-02	0,06	0,109	0,047
SASH1	6,11E-03	0,05	0,101	0,040
PSMG3	3,19E-02	0,05	0,101	0,043
CCND1	2,77E-02	-0,08	0,035	0,115
ENSGALG00000039028	4,66E-02	-0,08	0,037	0,116
CNTF	9,60E-03	-0,09	0,040	0,127
CDC42EP4	5,15E-03	-0,09	0,037	0,126
ENSGALG00000036498	2,88E-04	-0,09	0,013	0,102
SLC4A7	6,28E-05	-0,10	0,011	0,104
NDUFA1	4,44E-02	-0,10	0,133	0,243
CSTB	4,64E-02	-0,10	0,074	0,167
WNT4	1,58E-03	-0,10	0,019	0,103
ND1	4,81E-03	-0,10	0,705	0,861
SELENOF	9,80E-03	-0,10	0,064	0,160
PSMG4	8,97E-03	-0,10	0,064	0,160
APOA1	1,05E-03	-0,10	0,021	0,110
GSN	2,06E-03	-0,11	0,048	0,147
FERMT1	1,43E-05	-0,11	0,005	0,101
TNFRSF21	1,07E-04	-0,11	0,019	0,115
LIFR	6,50E-04	-0,11	0,027	0,120
ENSGALG00000013167	2,00E-05	-0,11	0,019	0,123
CD151	1,76E-03	-0,11	0,048	0,147
MXRA5	3,57E-03	-0,11	0,024	0,107
COX19	1,82E-02	-0,11	0,096	0,198
ENSGALG00000029544	3,05E-02	-0,12	0,136	0,244
ITGA6	6,18E-05	-0,12	0,016	0,113
PRDX3	6,99E-05	-0,12	0,069	0,192
FKBP5	1,59E-04	-0,12	0,027	0,126
SGK1	3,76E-05	-0,12	0,013	0,111
STOM	2,17E-03	-0,12	0,072	0,179
ENSGALG00000017357	2,08E-03	-0,12	0,085	0,195
TIMM10	4,28E-02	-0,12	0,122	0,224
WNT6	2,75E-05	-0,12	0,019	0,121
TRA2B	1,65E-03	-0,13	0,096	0,210
RPLP0	2,45E-02	-0,13	0,971	0,989
SNU13	1,04E-02	-0,13	0,194	0,315
ACTN1	8,02E-05	-0,13	0,056	0,172
GNAI2	5,82E-07	-0,13	0,013	0,129
NDUFS7	2,89E-02	-0,13	0,154	0,262
AIRC	9,70E-06	-0,13	0,048	0,170
TNIP2	7,92E-03	-0,13	0,051	0,143
CDK6	1,40E-07	-0,14	0,016	0,140
KRT15	1,20E-06	-0,14	0,016	0,131
ITGA3	1,94E-03	-0,14	0,082	0,191
RPL26	3,92E-03	-0,14	0,987	0,996
APP	9,03E-05	-0,14	0,074	0,198
ANP32B	2,52E-02	-0,14	0,205	0,320
ADSS	5,20E-08	-0,14	0,021	0,152
TNS1	5,45E-08	-0,14	0,019	0,148
ENSGALG00000041778	1,77E-07	-0,14	0,024	0,152
ENSGALG00000016324	1,15E-06	-0,14	0,013	0,126
cNFI-A	2,73E-04	-0,14	0,104	0,230

Supplementary Table S3 (continued). Gene expression levels in KRT9LC2-positive (differentiated, scale epidermis) versus KRT14L1-positive (non-differentiated) KC

Gene	P-value (adjusted)	Average Log(e) FC	pct.1	pct.2
NDUFAF8	2,95E-04	-0,14	0,104	0,227
ODC1	4,48E-05	-0,14	0,048	0,163
ENSGALG00000042629	3,88E-07	-0,14	0,051	0,190
NFIB	4,11E-05	-0,14	0,074	0,200
COX14	3,84E-05	-0,14	0,090	0,222
RPL31	1,91E-03	-0,14	0,952	0,980
REEP5	1,09E-03	-0,14	0,157	0,284
PDXK	2,72E-02	-0,14	0,160	0,267
COL5A1	6,82E-08	-0,15	0,011	0,133
NDUFB6	1,94E-03	-0,15	0,165	0,290
FMC1	4,97E-07	-0,15	0,048	0,183
TLCD1	2,95E-05	-0,15	0,098	0,232
ENSGALG00000037920	1,83E-05	-0,15	0,093	0,229
ENSGALG00000004701	5,66E-04	-0,15	0,215	0,352
RPL8	1,05E-05	-0,15	0,989	0,998
RPL4	2,47E-05	-0,15	0,987	0,991
RPS14	4,35E-05	-0,15	0,979	0,997
ENSGALG00000008620	2,19E-04	-0,15	0,976	0,988
RPLP1	2,05E-07	-0,15	0,997	0,999
TKT	2,15E-06	-0,16	0,098	0,246
NDUFB7	2,09E-04	-0,16	0,205	0,344
ATP5J	1,31E-02	-0,16	0,420	0,520
RPS15	1,69E-07	-0,16	1,000	1,000
MRPS12	3,77E-06	-0,16	0,136	0,289
NDUFB1	1,43E-04	-0,16	0,197	0,337
CD47	2,71E-04	-0,16	0,117	0,245
ENSGALG00000040297	4,80E-06	-0,16	0,101	0,244
MRPS21	1,05E-04	-0,16	0,194	0,335
PGAM1	3,21E-04	-0,17	0,215	0,350
COX7C	1,88E-02	-0,17	0,359	0,466
DNTTIP2	4,55E-05	-0,17	0,178	0,321
SUMO1	5,64E-07	-0,17	0,093	0,243
HMGN2	1,08E-02	-0,17	0,141	0,249
MRPL51	8,84E-07	-0,17	0,085	0,231
MINOS1	4,75E-04	-0,17	0,255	0,393
EIF1	1,02E-03	-0,17	0,745	0,791
ENSGALG00000036057	2,01E-07	-0,17	0,080	0,230
ENSGALG00000013072	3,15E-09	-0,17	0,024	0,168
ATP1A1	2,15E-10	-0,17	0,024	0,179
VDAC2	2,87E-04	-0,17	0,255	0,390
ASS1	1,15E-09	-0,17	0,035	0,188
ENSGALG00000000137	2,07E-04	-0,17	0,234	0,378
S100A6	1,54E-02	-0,17	0,878	0,951
PEBP1	1,75E-05	-0,18	0,191	0,342
EIF5	1,60E-03	-0,18	0,237	0,365
ENSGALG00000029172	7,47E-04	-0,18	0,319	0,446
CALM.1	1,57E-02	-0,18	0,426	0,520
ENSGALG00000036073	2,14E-05	-0,18	0,011	0,109
ENSGALG00000016471	2,65E-11	-0,18	0,029	0,195
SOD1	2,71E-10	-0,18	0,053	0,222
PTGS2	1,57E-06	-0,18	0,037	0,162
NSA2	5,20E-05	-0,18	0,245	0,388
MSN	6,66E-11	-0,18	0,045	0,219
PHYHIPL	3,03E-08	-0,18	0,093	0,257
SMIM4	1,10E-02	-0,18	0,420	0,516
ENSGALG00000028600	2,52E-06	-0,18	0,968	0,985
ENSGALG00000002771	1,06E-05	-0,19	0,098	0,236
COX5A	3,72E-04	-0,19	0,444	0,549
ENSGALG00000007936	7,13E-09	-0,19	0,146	0,330
COL17A1	5,00E-08	-0,19	0,029	0,164
ITGB1	2,10E-12	-0,19	0,019	0,189
ENSGALG00000030902	1,64E-03	-0,19	0,231	0,360
PHB2	4,97E-06	-0,19	0,205	0,360
CD99	1,38E-13	-0,19	0,019	0,199
COL3A1	3,67E-08	-0,19	0,011	0,136
ATPIF1	5,49E-04	-0,20	0,455	0,560
RPL11	4,58E-08	-0,20	0,915	0,985
CD81	4,19E-04	-0,20	0,492	0,608
RPL27	9,35E-08	-0,20	0,947	0,969
SLC25A3	6,78E-06	-0,20	0,322	0,476
ENSGALG00000030120	7,93E-05	-0,20	0,790	0,828
GLUL	6,82E-13	-0,20	0,027	0,206
ENSGALG00000001634	1,35E-15	-0,20	1,000	1,000
HSPE1	1,72E-08	-0,21	0,229	0,416
CHURC1	3,55E-05	-0,21	0,378	0,507
NDFIP1	3,84E-07	-0,21	0,258	0,424
SNRPD3	3,03E-08	-0,21	0,245	0,420
CALM	5,76E-06	-0,21	0,226	0,380
SVBP	1,78E-09	-0,21	0,170	0,359
RPL10A	2,05E-13	-0,21	0,981	0,996
RPL3	2,39E-10	-0,21	0,939	0,978
CAVIN1	8,94E-14	-0,22	0,045	0,241
ENSGALG00000001475	3,08E-06	-0,23	0,035	0,155
NCL	5,39E-06	-0,23	0,436	0,564
KRT5L1	5,22E-10	-0,23	0,027	0,180
IP6K2	1,89E-09	-0,23	0,125	0,301
ROMO1	2,18E-08	-0,23	0,282	0,468
YWHAQ	9,12E-08	-0,23	0,386	0,551
ENSGALG00000006805	6,28E-10	-0,23	0,074	0,248
ENSGALG00000031853	1,14E-08	-0,23	0,274	0,455
ATP5J2	7,08E-09	-0,23	0,354	0,535
SEC61G	2,56E-12	-0,24	0,178	0,395
COX6B1	9,21E-08	-0,24	0,500	0,630

Supplementary Table S3 (continued). Gene expression levels in KRT9LC2-positive (differentiated, scale epidermis) versus KRT14L1-positive (non-differentiated) KC

Gene	P-value (adjusted)	Average Log(e) FC	pct.1	pct.2
ENSGALG00000037017	3,50E-09	-0,24	0,306	0,486
SEPP1	1,41E-07	-0,25	0,266	0,435
UQCRQ	3,82E-13	-0,25	0,138	0,353
COL1A2	9,80E-12	-0,25	0,021	0,188
ENSGALG00000002932	3,79E-17	-0,25	0,931	0,975
RPL22	3,94E-15	-0,25	0,883	0,957
FAM213A	2,66E-12	-0,26	0,090	0,286
RPL23A	8,06E-20	-0,26	0,989	0,996
ENSGALG00000016691	2,22E-08	-0,26	0,566	0,700
SNRPD1	1,31E-16	-0,26	0,128	0,373
COX4I1	4,45E-11	-0,27	0,487	0,641
RPL9	5,19E-17	-0,27	0,952	0,994
ENSGALG00000007863	3,14E-12	-0,27	0,322	0,519
POSTN	4,96E-14	-0,27	0,035	0,226
ATP1B3	8,63E-13	-0,27	0,287	0,503
ENSGALG00000035996	8,07E-15	-0,27	0,202	0,438
RPL21	5,45E-27	-0,28	0,997	1,000
ENSGALG00000004509	1,94E-06	-0,28	0,976	0,983
RPL34	8,48E-20	-0,28	0,955	0,992
RPS12	8,56E-17	-0,28	0,995	0,992
PPIB	1,45E-13	-0,28	0,316	0,532
RPS16	6,53E-18	-0,29	0,928	0,984
ENSGALG00000042011	1,41E-12	-0,29	0,431	0,612
GAPDH	4,22E-16	-0,29	0,822	0,922
CLU	5,03E-19	-0,29	0,053	0,294
CHCHD2	1,28E-11	-0,30	0,612	0,705
ENSGALG00000006899	5,01E-14	-0,30	0,388	0,603
RPL13	2,18E-22	-0,30	0,957	0,990
ENSGALG00000026970	1,06E-17	-0,30	0,040	0,263
DDX5	2,84E-12	-0,30	0,468	0,627
UBA52	2,37E-20	-0,31	0,931	0,968
ATP5G3	5,05E-15	-0,31	0,375	0,584
ENSGALG00000031593	1,58E-23	-0,31	0,019	0,280
S100A11	1,79E-14	-0,31	0,814	0,921
RPL19	4,44E-25	-0,31	0,941	0,994
ENSGALG00000043379	1,55E-27	-0,31	0,960	0,997
SEPW1	1,94E-20	-0,32	0,311	0,572
EIF4EBP1	3,35E-15	-0,32	0,444	0,627
RPSA	3,67E-34	-0,34	0,960	0,996
RPL30	2,05E-29	-0,35	0,957	0,993
ENSGALG00000017380	2,67E-23	-0,35	0,215	0,500
ITM2B	1,12E-21	-0,36	0,176	0,448
YBX1	6,44E-22	-0,36	0,630	0,829
ENSGALG00000001250	7,93E-28	-0,36	0,112	0,428
MGP	5,05E-18	-0,37	0,021	0,241
HSPB1	1,92E-26	-0,37	0,029	0,317
PRDX1	2,67E-23	-0,38	0,205	0,498
ATP5I	3,23E-25	-0,38	0,194	0,495
RPL32	3,06E-41	-0,38	0,992	0,998
FAU	2,15E-41	-0,38	0,979	0,997
ENSGALG00000002286	1,31E-23	-0,38	0,822	0,909
RPS17	3,23E-37	-0,39	0,987	0,999
COX7A2	1,34E-27	-0,39	0,210	0,523
FOS	1,72E-04	-0,40	0,662	0,747
RPS3	6,39E-36	-0,40	0,912	0,986
ITM2A	1,45E-23	-0,41	0,455	0,677
H2AFV	1,43E-21	-0,42	0,324	0,572
ATIC	4,33E-34	-0,43	0,082	0,432
ATP5G1	6,45E-30	-0,44	0,346	0,633
COL14A1	5,34E-35	-0,46	0,048	0,397
RPL38	6,90E-39	-0,47	0,710	0,927
S100A10	6,56E-31	-0,48	0,019	0,334
RPS25	1,56E-52	-0,48	0,949	0,993
RPL37A	1,33E-49	-0,48	0,976	0,996
DCN	1,12E-41	-0,49	0,029	0,415
ENSGALG00000023818	1,60E-19	-0,54	0,713	0,883
SPARC	1,51E-38	-0,55	0,085	0,462
UBC	6,19E-43	-0,55	0,630	0,845
RPS23	3,07E-53	-0,56	0,883	0,966
BSG	1,73E-51	-0,57	0,045	0,488
DST	5,23E-41	-0,60	0,056	0,449
ENSGALG00000007220	1,81E-51	-0,61	0,231	0,667
RPS28	1,08E-67	-0,62	0,875	0,983
NPM1	8,24E-60	-0,63	0,348	0,757
Wpkci-8	4,13E-46	-0,63	0,301	0,678
SAT1	1,09E-43	-0,65	0,287	0,655
RPS27	2,37E-65	-0,67	0,947	0,989
RPS4	8,90E-69	-0,67	0,928	0,992
ENSGALG00000004952	1,03E-73	-0,74	0,838	0,982
ENSGALG00000026383	2,52E-74	-0,80	0,112	0,659
RPL36	9,40E-67	-0,85	0,779	0,942
RPL37	4,51E-72	-0,86	0,854	0,981
RPL29	6,47E-83	-0,87	0,803	0,985
RPS21	7,33E-82	-1,01	0,574	0,938
ENSGALG00000012229	9,20E-78	-1,13	0,580	0,902
KRT14L1	2,25E-213	-1,56	0,189	1,000

Abbreviations: FC, fold change; pct, percent; KC, keratinocytes
